# Surface Cross-Linking by Macromolecular Tethers Enhances Virus-like Particles’ Resilience to Mucosal Stress Factors

**DOI:** 10.1021/acsnano.3c10339

**Published:** 2024-01-18

**Authors:** Ahmed Ali, Suwannee Ganguillet, Yagmur Turgay, Timothy G. Keys, Erika Causa, Ricardo Fradique, Viviane Lutz-Bueno, Serge Chesnov, Chia-Wei Tan-Lin, Verena Lentsch, Jurij Kotar, Pietro Cicuta, Raffaele Mezzenga, Emma Slack, Milad Radiom

**Affiliations:** † Department of Health Sciences and Technology, ETH Zürich, Zürich 8092, Switzerland; ‡ Biological and Soft Systems, Cavendish Laboratory, University of Cambridge, Cambridge CB3 0HE, U.K.; § Paul Scherrer Institute (PSI), Villigen 5232, Switzerland; ∥ Laboratoire Léon Brillouin, CEA-CNRS (UMR-12), CEA Saclay, Université Paris-Saclay, Gif-sur-Yvette Cedex 91191, France; ⊥ Functional Genomics Centre Zürich (FGCZ), University of Zürich/ETH Zürich, Zürich 8057, Switzerland

**Keywords:** virus-like particle vaccines, mucosal delivery, nanoindentation, mucus interactions, polyethylene glycol tethers, biomedical applications

## Abstract

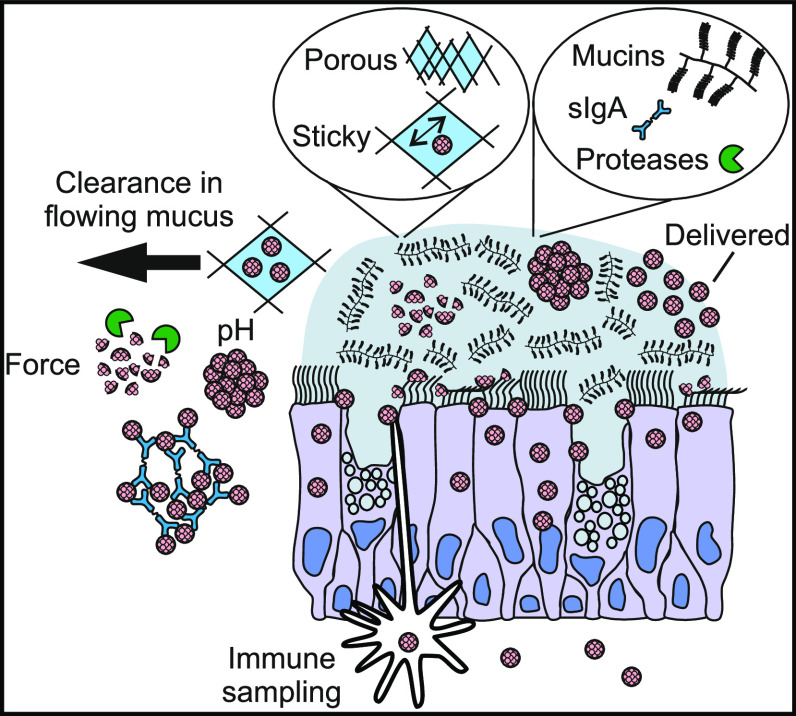

Virus-like particles (VLPs) are emerging as nanoscaffolds in a variety of biomedical applications including delivery of vaccine antigens and cargo such as mRNA to mucosal surfaces. These soft, colloidal, and proteinaceous structures (capsids) are nevertheless susceptible to mucosal environmental stress factors. We cross-linked multiple capsid surface amino acid residues using homobifunctional polyethylene glycol tethers to improve the persistence and survival of the capsid to model mucosal stressors. Surface cross-linking enhanced the stability of VLPs assembled from *Acinetobacter* phage AP205 coat proteins in low pH (down to pH 4.0) and high protease concentration conditions (namely, in pig and mouse gastric fluids). Additionally, it increased the stiffness of VLPs under local mechanical indentation applied using an atomic force microscopy cantilever tip. Small angle X-ray scattering revealed an increase in capsid diameter after cross-linking and an increase in capsid shell thickness with the length of the PEG cross-linkers. Moreover, surface cross-linking had no effect on the VLPs’ mucus translocation and accumulation on the epithelium of *in vitro* 3D human nasal epithelial tissues with mucociliary clearance. Finally, it did not compromise VLPs’ function as vaccines in mouse subcutaneous vaccination models. Compared to PEGylation without cross-linking, the stiffness of surface cross-linked VLPs were higher for the same length of the PEG molecule, and also the lifetimes of surface cross-linked VLPs were longer in the gastric fluids. Surface cross-linking using macromolecular tethers, but not simple conjugation of these molecules, thus offers a viable means to enhance the resilience and survival of VLPs for mucosal applications.

Virus-like particles (VLPs) have emerged as invaluable nanocarriers in biomedical applications.^1,2^ Sensitive cargo can be protected by packaging it inside their capsids while genetic and/or chemical modifications can be used to deliver vaccine antigens, or to target the cargo, to specific cell types. In recent years, various therapeutic molecules have been integrated into VLPs for immunotherapy,^3^ gene therapy,^4−6^ chemotherapy,^7−9^ as well as for contrast imaging and photothermal therapy.^10,11^

As carriers, VLPs must endure environmental challenges and it is likely that their material properties are an important factor determining their fate.^12^ General key considerations for “delivery-capable” VLPs include size, colloidal stability, target specificity, and responsiveness to stimuli for cargo release. For mucosal applications, VLPs are faced with mucosal-specific physiological challenges including a thick and adhesive mucus gel, mucus clearance from the site of delivery (e.g., via mucociliary clearance in respiratory epithelia or peristalsis and fecal stream in intestinal epithelium), high concentrations of proteases and secretory immunoglobulin A (sIgA) antibodies and intermittent mechanical agitation.^13−15^ Together these effects can significantly reduce the efficacy and survival of VLPs for mucosal applications (Figure 1(a)).^16^

**Figure 1:**
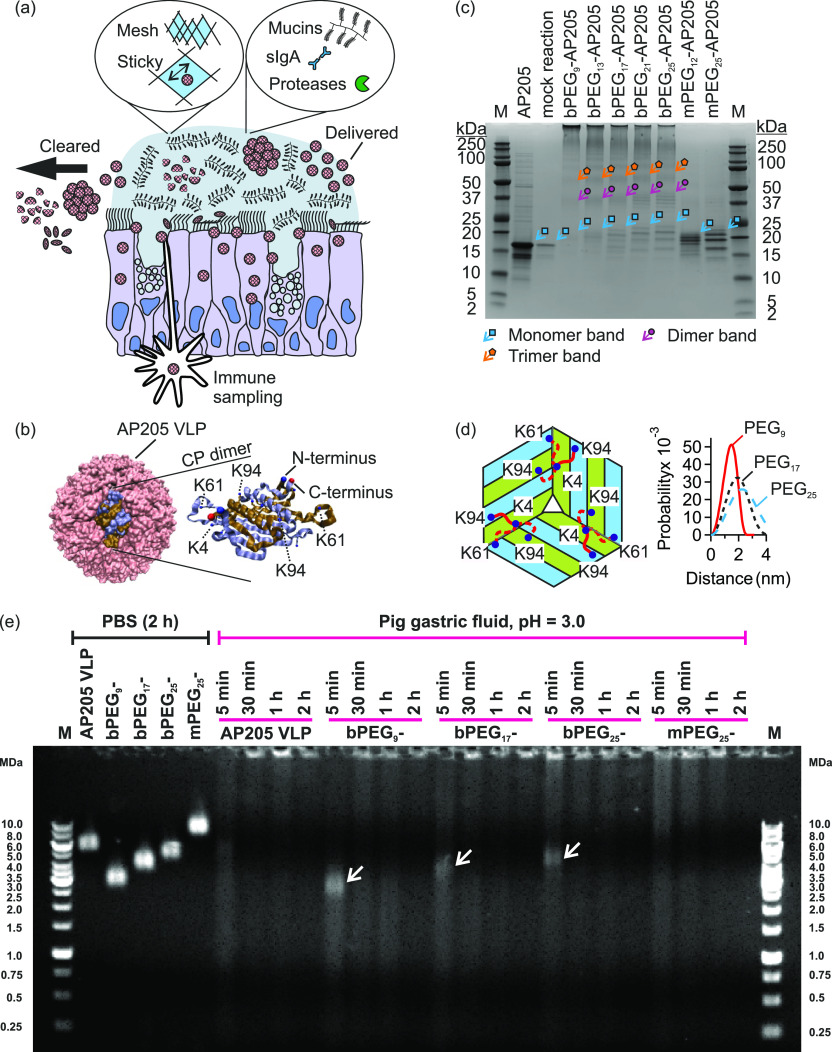
Susceptibility of virus-like particles (VLPs) to mucosal factors, and their capsid stabilization by means of surface cross-linking using macromolecular tethers. (a) VLPs are exposed to physical (e.g., mechanical agitation, pH, temperature, and anatomical clearance mechanisms) and biochemical challenges (e.g., sIgA and proteases) on mucosal surfaces which reduce their stability and survival. (b) Schematic of AP205 VLP and schematic of one coat protein (CP) dimer.^42^ The N-terminus (blue) of one monomer, the C-terminus (red) of the other monomer, together with several lysine residues are marked. (c) Reducing SDS-PAGE of native AP205 VLP, AP205 VLP in mock reaction (i.e., in the presence of DMSO), PEG-cross-linked (bPEG_*n*_-, *n* = 9–25), and PEGylated but not cross-linked AP205 VLPs (mPEG_*n*_-, *n* = 12 or 25). M is PageRuler Plus Prestained Protein Ladder. The monomer region is marked with cyan arrows/squares, dimer region with purple arrows/circles, and trimer region with orange arrows/pentagons. (d) Schematic of AP205 CP arrangement near the 3-fold axis of symmetry and the assignment of lysine residues K4, K61, and K94 that participated in the cross-linking reaction. In the schematic, three dimers are shown with their monomers colored in blue and green. Only one tether (red coil) connected either of the two of K4–K94 linkage possibilities or the one of K4–K61 linkage possibility. The end-to-end probability distributions of bPEG_*n*_ tethers (*n* = 9, 17 and 25) calculated from an analytical worm-like chain model.^44^ (e) Agarose gel electrophoresis of native AP205 VLP, bPEG_9_-AP205 VLP, bPEG_17_-AP205 VLP, bPEG_25_-AP205 VLP, and mPEG_25_-AP205 VLP in PBS and in pig gastric fluid (pH 3.0), respectively, on the left and right of the gel. Incubation times varied between 5 min, 30 min, 1 h, and 2 h at 37 °C. Arrows at time point 5 min indicate persistent PEG-cross-linked AP205 VLPs in gastric fluid. M stands for marker.

Conjugation of macromolecules (without cross-linking) to VLP or viral vector capsids has been exploited as a means to enhance biological stability and function. For example, polyethylene glycol conjugation (PEGylation) protected adenovirus vector (Adv) from neutralizing antibodies, prolonged transgene expression and reduced immune activation against the vector.^17−19^ Subsequent experiments revealed a complex dependence on the biophysicochemical properties of the conjugated product.^20−22^ Thereby, for example, optimization of the conjugation conditions and the molecular weight of the PEG molecules further reduced antivector serum antibody and increased the persistence of transgene expression.^23^ Other macromolecules were conjugated to bacteriophage Qβ VLP to shield it from immune recognition.^24,25^ In other directions, PEG was used to tether a fluorescent molecule or folic acid to cowpea mosaic virus for intravital vascular imaging and for targeted cargo delivery to cancer cell lines, respectively.^26,27^

Previous investigations suggest that conjugation of a synthetic polymer to the capsid surface increases colloidal stability, mainly due to excluded volume interactions with serum biomolecules (including antibodies and complement system) and nonspecific interactions with cell receptors. There is no compelling evidence regarding other aspects of stability, e.g., in the face of intermittent mechanical agitation and shear forces, and/or in exposure to high concentrations of digestive enzymes. These are generally characteristics of mucosal tissues, so-called barrier functions. For a rational use of protein cages for mucosal applications, these stability criteria require further investigations. To this end, there is compelling evidence that viruses, viral vectors, and VLPs disintegrate under mechanical force^28−37^ and under unfavorable chemical environments.^38,39^

Our model VLP was assembled from 180 copies of *Acinetobacter* phage AP205 coat protein.^37^ AP205 VLP is a versatile platform for genetic and/or chemical conjugation of various antigenic molecules to capsid surface due to surface exposed N- and C-termini and has therefore potentiated several vaccination strategies.^37,40−42^ We investigated the effects of PEGylation using monofunctional PEG linkers and using homobifunctional PEG cross-linkers, the latter resulting in cross-linking of AP205 VLP multiple capsid surface residues (namely the lysine residues). Surface cross-linking increased the stability of the VLPs to low pH and high protease concentration conditions and further enhanced their resistance to mechanical agitation. Additionally, PEG surface cross-linking did not affect the translocation of VLPs across mucus in *in vitro* 3D human nasal epithelium with mucociliary clearance. Furthermore, PEG cross-linking did not shield immune recognition of capsid coat proteins, therefore not inhibiting function as a vaccine in mouse subcutaneous vaccination models. Surface cross-linking using macromolecular tethers thus offers a viable means to enhance the resilience and survival of VLPs in mucosal environments.

## Results and Discussion

The sequence of AP205 monomer and the details of PEG molecules are presented in S1. Surface cross-linked and simply PEGylated VLPs were produced using homobifunctional and monofunctional PEG molecules, the resulting VLPs are respectively named bPEG_*n*_- and mPEG_*n*_-AP205 VLP, where *n* is the number of PEG monomers. We targeted lysine residues for cross-linking as these are available on capsid surface and are in proximity for cross-linking (cf. section below). We therefore selected *N*-hydroxysuccinimide (NHS) ester as the functional group of the PEG molecules requiring mild reaction conditions and purification steps (cf. Figure 1(b), and Materials and Methods section).

### Surface Cross-Linking of AP205 VLP Capsid Using Polyethylene Glycol (PEG) Macromolecular Tethers

Under the conditions used, the cross-linking reaction was at the level of single VLP particles, and we did not observe aggregation between the particles using dynamic light scattering (DLS) (Figure S2–1 and Figure S2–2). Transmission electron microscopy (TEM) showed that the VLPs retained their spherical geometry after the reaction and no detectable size variation for any lengths of PEG cross-linker, *n* = 9–25, was observed (Figure S3–1).

Investigations with reducing SDS-polyacrylamide gel electrophoresis (SDS-PAGE) revealed that reaction with homobifunctional PEG cross-linkers resulted in PEG-cross-linked AP205 dimers, trimers, and possibly higher oligomers. In the representative SDS-PAGE shown in Figure 1(c), in the lanes associated with bPEG_*n*_-AP205 VLPs (*n* = 9 to 25), bands in the MW range 37–50 kDa (marked with purple arrows/circles) correspond to PEG-cross-linked AP205 dimers, and bands in MW range 50–75 kDa (marked with orange arrows/pentagons) correspond to PEG-cross-linked AP205 trimers. Densitometry profiles of the reducing SDS-PAGE in Figure 1(c) clearly showed the MW bands associated with AP205 dimers, trimers, tetramers, pentamers and higher order oligomers (Figure S4–1). These bands were absent in the mock reaction and in mPEG_25_-AP205 VLP. Electrospray ionization mass spectroscopy (ESI-MS) revealed that in addition to conjugation from both ends (cross-linking), up to four PEGs per coat protein conjugated from only one end to the coat proteins (S5).

Using nano ultraperformance liquid chromatography coupled to ESI-MS, we then investigated the lysine (K) residues that were involved in the cross-linking reaction. It was found that K4 and K61 and K4 and K94 were cross-linked (Table S5–7, cf. Figure 1(b)). In Figure 1(d), a schematic of PEG linkages between individual AP205 monomers near the 3-fold axis of symmetry of the capsid is shown. Using coordinates from cryoelectron microscopy reconstruction of the AP205 VLP (Protein Data Bank ID: 5LQP) in Visual Molecular Dynamics (VMD),^42,43^ the N to N interatomic distance in K4–K61 linkage was estimated to be 1.8 nm and in K4–K94 linkage to be 1.6 and 2.4 nm. Calculation of end-to-end probability distributions of PEG_9_, PEG_17_, and PEG_25_ linker molecules using worm-like chain model are shown in Figure 1(d).^44^ The calculated extension lengths were consistent with the interatomic distances; however, lengthier molecules reached greater distances with a higher probability. Additional details regarding the conjugation stoichiometry, mass spectroscopy, and SDS-PAGE are found in S2, S4, and S5, respectively.

### Enhanced Colloidal and Enzymatic Stability of Surface-Cross-Linked AP205 VLPs

VLPs are potentially interesting scaffolds for the oral or intranasal delivery of biomolecules, requiring stability in pH-varying environments and in the presence of high concentrations of proteases.^1,2,12^ Therefore, increasing the colloidal and enzymatic stability in the complex mucosal environment is of high translational relevance.

Experiments using DLS showed that native VLP was stable at pH 7.4 but aggregated at all pH from 6.0 to 2.0 (Figure S6–1). PEG-cross-linking increased the stability of the VLPs down to pH ∼ 4.0 (Figure S6–2).

Using agarose gel electrophoresis, we then tested the enzymatic stability of PEG-cross-linked VLPs in pig and mouse gastric fluids. In PBS, an incubation time of 2 h (or longer) at 37 °C did not affect the stability or electrophoretic mobility of the VLPs^37^ (Figure 1(e)). In pig gastric fluid, which had a pH of 3.0 and a high protease concentration, PEG-cross-linked VLPs, namely bPEG_9_-AP205 VLP, bPEG_17_-AP205 VLP, and bPEG_25_-AP205 VLP, showed improved stability after 5 min of incubation. In particular, a band, marked with an arrow, remained at the expected MW, with fewer breakdown products than the native VLP. At the later time points of 30 min, 1 and 2 h, the intact portion was disintegrated, and signs of aggregation and accumulation of material in the wells of the gel appeared. Interestingly, the PEGylated but not cross-linked VLP, namely mPEG_25_-AP205 VLP, disintegrated earlier compared to bPEG_25_-AP205 VLP.

Experiments in pig gastric fluid at pH 4.7 corroborated the stabilizing effect of PEG-cross-linking in the presence of proteases (Figure S7–1). At this pH, the PEG-cross-linked VLPs were stable during longer periods. In pig gastric fluid at pH 5.5, all VLPs were found to be stable over 1.5 h of incubation (Figure S7–2). Incubation in mouse gastric fluid at pH 4.6 presented a similar behavior showing the stabilizing effect of PEG modification (Figure S7–3).

An additional experiment was performed to estimate the disintegration rates of the VLPs. In this case, after each incubation period with pig gastric fluid at pH 3.0, the VLP solution was mixed with a solution containing protease inhibitor and frozen on dry ice (Figure S7–4). It was found that PEG-cross-linking noticeably reduced the disintegration rate compared with the native VLP. A comparison of the destabilization rates between bPEG_25_-AP205 VLP and mPEG_25_-AP205 VLP revealed the stabilizing effect of cross-linking. In both cases, the PEG-modified VLPs were degraded because the number density of PEG molecules on VLP surface was low, allowing protease access. Despite protease cleavage, the capsid of PEG-cross-linked VLPs persisted longer because the covalently linked lysine residues remained connected.

The DLS and agarose gel electrophoresis observations indicated that PEG-cross-linking of VLP capsid, but not one-end conjugation of PEGs, is a viable approach to overcome the susceptibility of VLPs to enzymatic instability.

### Enhanced Stiffness of Surface-Cross-Linked AP205 VLPs

Susceptibility to mechanical stress sets a limit on potential applications of VLPs in various mucosal tissues. Mechanical agitation occurs during interactions with cells, e.g., during endocytosis or antigen presentation to B cells, in proximity to beating cilia or when sheared against the fecal stream.^45−51^ Using different force probing techniques, the endocytosis “pulling” force exerted by a cell onto a virus, B cell antigen extraction, and the force of cilia agitation were measured to vary between a few to hundreds of pN.^45−51^ Although this force range is generally below the yield force values of VLPs, when applied repeatedly as they do *in vivo*, they can disintegrate the capsids due to fatigue.^28−30,34−37^ As a result, enhancing the mechanical stability in the mucosal environment holds substantial translational relevance.

**Figure 2:**
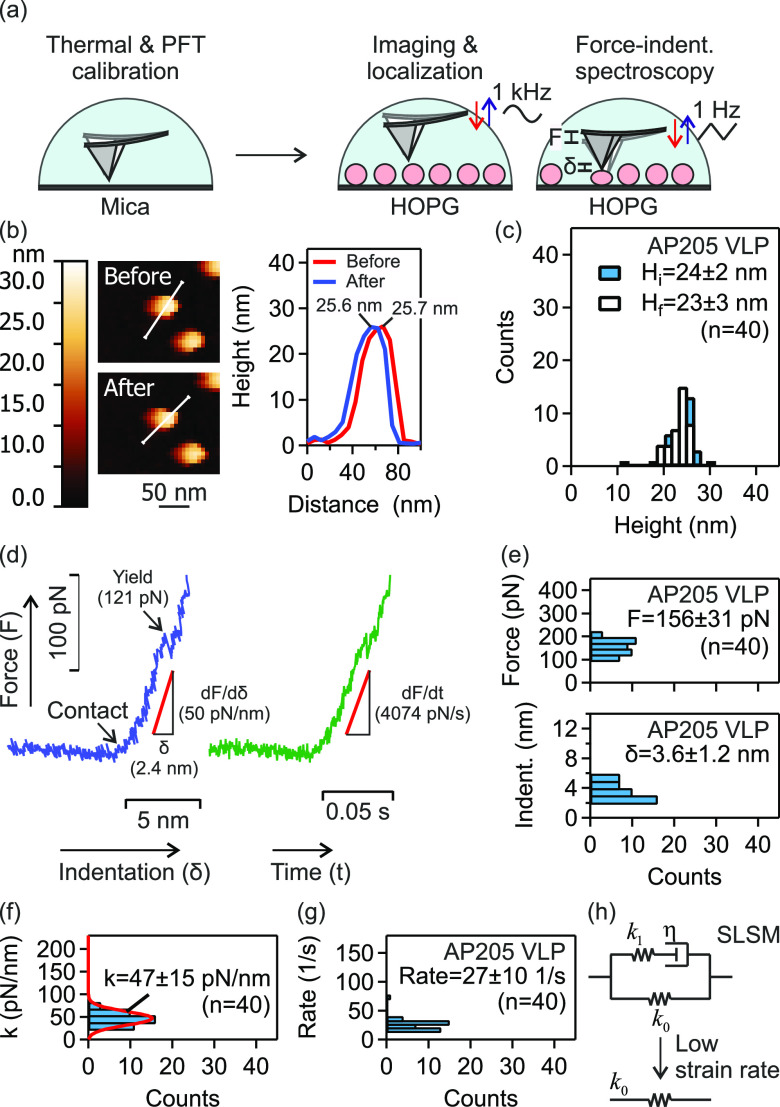
Mechanical properties of AP205 VLP investigated using AFM force–indentation spectroscopy. (a) Schematic of AFM calibration on freshly cleaved mica, and force–indentation spectroscopy of HOPG-immobilized VLPs. Single VLPs were localized using PeakForce tapping imaging (base tapping rate of 1 kHz) prior to force measurements using normal ramp at base approach-retraction rate at 1 Hz. (b) Example of height evaluation for a single AP205 VLP before and after force application. (c) Height distributions of AP205 VLP before and after force measurements. (d) Example of force–indentation distance and force–indentation time curves for AP205 VLP. The linear regime of force–indentation distance response was used to calculate the stiffness. In the same range, a linear fit to force–indentation time response gave the indentation force rate. (e–g) Histograms of maximal force and indentation (indent.) (e), stiffness (f), and strain rate (g) of AP205 VLP. Mean ± standard deviation is reported. The number (*n*) of AP205 VLPs in repeat measurements is indicated. (h) Standard linear solid model (SLSM). At low strain rates, there is no contribution from friction (or rate dependent resistance) to indentation.

We examined the nanomechanical properties of native and PEG-cross-linked VLPs using atomic force microscopy (AFM) force–indentation spectroscopy. The schematic of AFM measurements is depicted in Figure 2(a) and details are provided in the Materials and Methods. The example shown in Figure 2(b) depicts a single representative AP205 VLP for which the height prior to indentation (*H*_i_) and after indentation (*H*_f_) were evaluated. Figure 2(c) shows the heights distributions of AP205 VLP with the respective average values. The force versus indentation distance (*F* – δ) curve was used to calculate the stiffness, and the force versus indentation time (*F* – *t*) curve to calculate the strain rate. An example of these curves for the same VLP (presented in Figure 2(b)) is shown in Figure 2(d). The linear part of the (*F* – δ) response was used to calculate the stiffness, *k* = d*F*/dδ, and the total indentation, δ = Δ*F*/*k*. The linear part of the (*F* – *t*) response was used to obtain the indentation force rate *Ḟ* = d*F*/d*t*, which was then converted to strain rate via γ = *Ḟ*/*kδ*.

After an initial linear response, the VLP yielded at a threshold force/indentation. The histograms of maximal force and indentation (indent.) of AP205 VLP together with the average values of these parameters are shown in Figure 2(e). The physical significance of these parameters is that at a force (or indentation) higher than the average maximal force (or indentation), yield (plastic deformation) occurs. After yielding, the original stiffness of the VLP will be significantly reduced.^28−30,34−37^

The histograms of stiffness and strain rate of AP205 VLP together with the average values of these parameters are shown in Figure 2(f) and (g), respectively. For this VLP, we previously showed that the standard linear solid model (SLSM, Figure 2(h)) interprets the stiffness as a function of the strain rate.^37^ We found that at a strain rate < 100 1/s, the overall mechanical response is governed by *k*_0_ in SLSM model. We retrieved a good agreement between the current evaluation of AP205 stiffness (47 ± 15 pN/nm) with the previous one (53 ± 23 pN/nm) at low strain rates.

**Figure 3:**
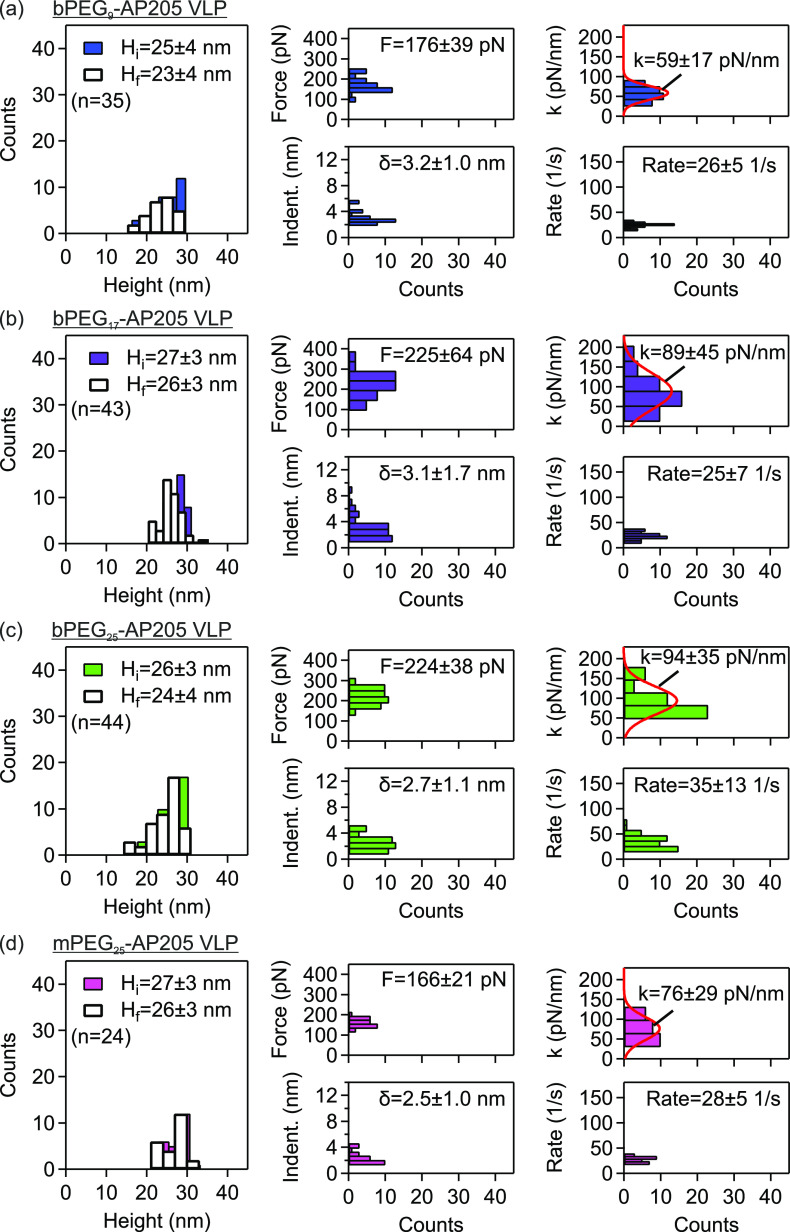
Enhanced strength and stiffness of PEG-cross-linked AP205 VLPs. Mechanical properties of PEG-cross-linked AP205 VLPs, namely bPEG_9_-AP205 VLP, bPEG_17_-AP205 VLP, and bPEG_25_-AP205 VLP, and PEGylated but not cross-linked mPEG_25_-AP205 VLP investigated using AFM force–indentation spectroscopy. Distributions of height before and after indentation, maximal force/indentation in linear response regime, and stiffness/strain rate are shown together with mean ± standard deviation. The number (*n*) of VLPs in repeat measurements is indicated.

Similarly, the mechanical properties of PEG-cross-linked VLPs, namely bPEG_9_-AP205 VLP, bPEG_17_-AP205 VLP, bPEG_25_-AP205 VLP were evaluated, and the results are shown in Figure 3(a–c), respectively. The initial heights of these VLPs were slightly higher than the native VLP. However, similar to the native VLP, the change from the initial height to the final height (i.e., after a single indentation, cf. Materials and Methods) was minute. The distributions of maximal force and indentation (indent.) showed variations compared to the native VLP. The average maximal force was found to be higher and accordingly the average maximal indentation lower compared to the native VLP. The increased (decreased) maximal force (indentation) of PEG-cross-linked VLPs potentially indicate reduced susceptibility to mechanical agitation. The distributions of stiffness and strain rate are also shown in the same figure. Accordingly, the average stiffnesses of these VLPs were also higher, by a factor of 1.3 to 2.0, compared to the stiffness of the native VLP at a similar range of strain rate.

For bPEG_9_-AP205 VLP, at a strain rate of about 10^4^ 1/s we previously found a stiffness of 46 ± 10 pN/nm.^37^ Our current measurement gave a stiffness of 59 ± 17 pN/nm at strain rate of 26 ± 5 1/s. The proximity of these values obtained at vastly different strain rates suggests that surface cross-linking changes the dynamic (or rate-dependent) mechanical response of the capsid. This behavior change could potentially be associated with the reduction of positive charges on capsid surface upon cross-linking of lysine residues as well as the introduction of PEG tether extension/relaxation (entropic spring) effects.

Additionally, the mechanical properties of PEGylated but not cross-linked AP205 VLP, namely mPEG_25_-AP205 VLP, were evaluated, and the results are shown in Figure 3(d). The initial height of mPEG_25_-AP205 VLP was higher than the native VLP, while the change from the initial height to height after a single indentation was small. The average maximal force and the average maximal indentation of mPEG_25_-AP205 VLP were respectively higher and lower compared to the native VLP. The average stiffness of this VLP was accordingly found to be higher than the native AP205, however smaller than the surface cross-linked AP205 VLP with similar number of PEG monomers, namely bPEG_25_-AP205 VLP (Figure 3(c)). Assuming similar effect from the reduction of positive charges due to lysine residues conjugation, the higher stiffness of bPEG_25_-AP205 VLP potentially indicates PEG chain stretching during indentation.

We make a note of a noticeable scattering in the stiffness data (cf. histograms of Figure 2(f) and Figure 3). Single-particle indentation of viruses and VLPs has often yielded large standard deviations. This variability can be attributed in part to potential misalignment of AFM tip on particle apex, given the comparable dimensions of the AFM tip diameter (e.g., 16 nm in AFM tips used in this work) and particle size (about 30 nm in diameter). Such misalignment has been demonstrated to lead to deviations in measured stiffness.^52^ The standard deviations are further influenced by variations in local nanomechanical properties of viruses and VLPs; the icosahedral geometry contains multiple axes of symmetries (e.g., 3-fold and 5-fold axis of symmetries in the case of AP205 VLP investigated here^42^). These different localities have been shown to exhibit distinct nanomechanical properties.^53^ Additionally, in our experiments, the PEG-modified VLPs were absorbed onto hydrophobic Highly Ordered Pyrolytic Graphite (HOPG). The hydrophilic PEG modification could destabilize the adhesion of the modified VLP onto HOPG, potentially resulting in particle rolling during indentation.

**Figure 4:**
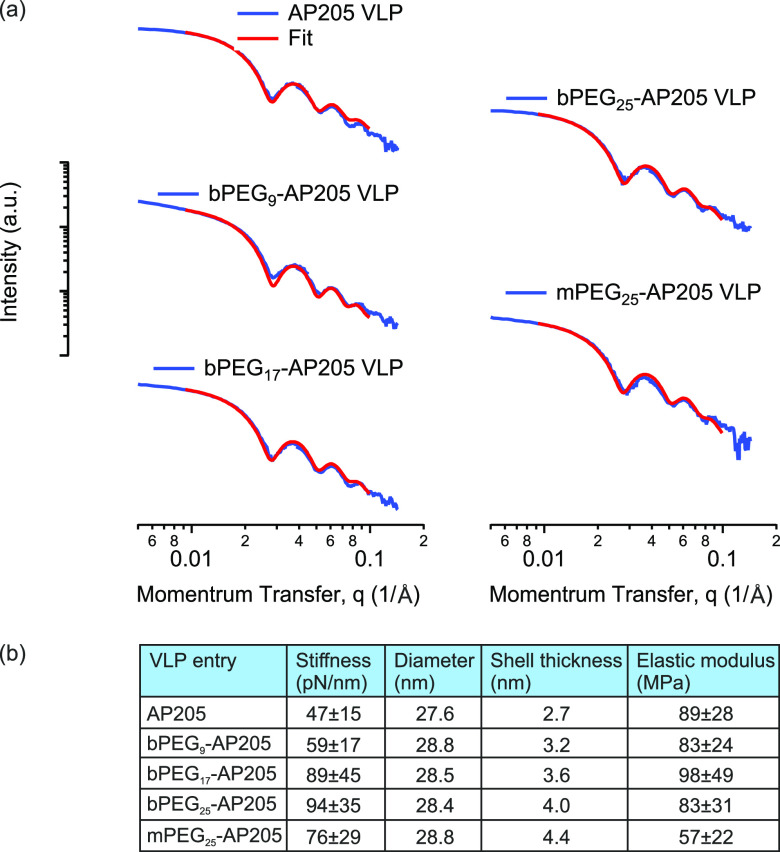
Geometrical and mechanical parameters of native and PEGylated AP205 VLPs. (a) Small angle X-ray scattering (SAXS) spectra of AP205, bPEG_9_-AP205, bPEG_17_-AP205, bPEG_25_-AP205, and mPEG_25_-AP205 VLPs, together with fits to spherical core–shell form factor. (b) Summary panel of the geometrical and mechanical properties of the VLPs.

We then evaluated capsid diameter and shell thickness using small-angle X-ray scattering (SAXS). The X-ray scattering spectra of the native and PEGylated VLPs together with fits to spherical core–shell form factor are shown in Figure 4(a). In each case, we found a good agreement based on the low values for the fitting error (χ^2^). In the summary panel in Figure 4(b), it is shown that the diameter (*D*) and shell thickness (*h*) of VLP increased after PEGylation. In addition, while the VLP diameter remained somewhat constant between the different lengths of PEG tethers, the shell thickness increased with PEG length and had its highest value for the PEGylated but not cross-linked VLP. Subsequent to these evaluations, the elastic modulus was calculated from the approximate relation *E* = *Dk*/2*h*^2^, Figure 4(b).^54,55^ The elastic moduli remained somewhat constant ∼90 MPa among the native and PEG-cross-linked VLPs. This observation can potentially be explained as follows. In nanoindentation experiments, the shell compression and bending moduli contribute to an effective elastic modulus *E*.^52,55,56^ Tethered PEG molecules disfavor local bending of the VLP particle because it results in chain stretching,^57^ therefore increasing the bending modulus. Reduction in coat protein positive surface charges after PEG conjugation reduces the interatomic repulsion during compression, therefore decreasing the compression modulus. Accordingly, the PEGylated but not cross-linked VLP, namely mPEG_25_-AP205 VLP, gave the lowest elastic modulus from the absence of chain stretching.

### Surface-Cross-Linking Does Not Restrict Mucus Translocation of AP205 VLPs in *In Vitro* 3D Human Nasal Epithelium with Mucociliary Clearance

A major feature of intestinal and respiratory tissue, where VLP application is relevant, is the production of mucus. Mucus is an adhesive and viscoelastic gel that covers the epithelium and functions to trap and dispose of foreign objects via various clearance mechanisms. In the airways, mucus is constantly pushed out via mucociliary clearance.^58,59^ We used *in vitro* 3D human nasal epithelial tissues to investigate the effect of PEG-cross-linking on translocation across flowing mucus. The tissues were characterized as having fully differentiated mucus producing goblet cells, ciliated cells with motile cilia, as well as other cells of normal human nasal epithelial tissue.

**Figure 5:**
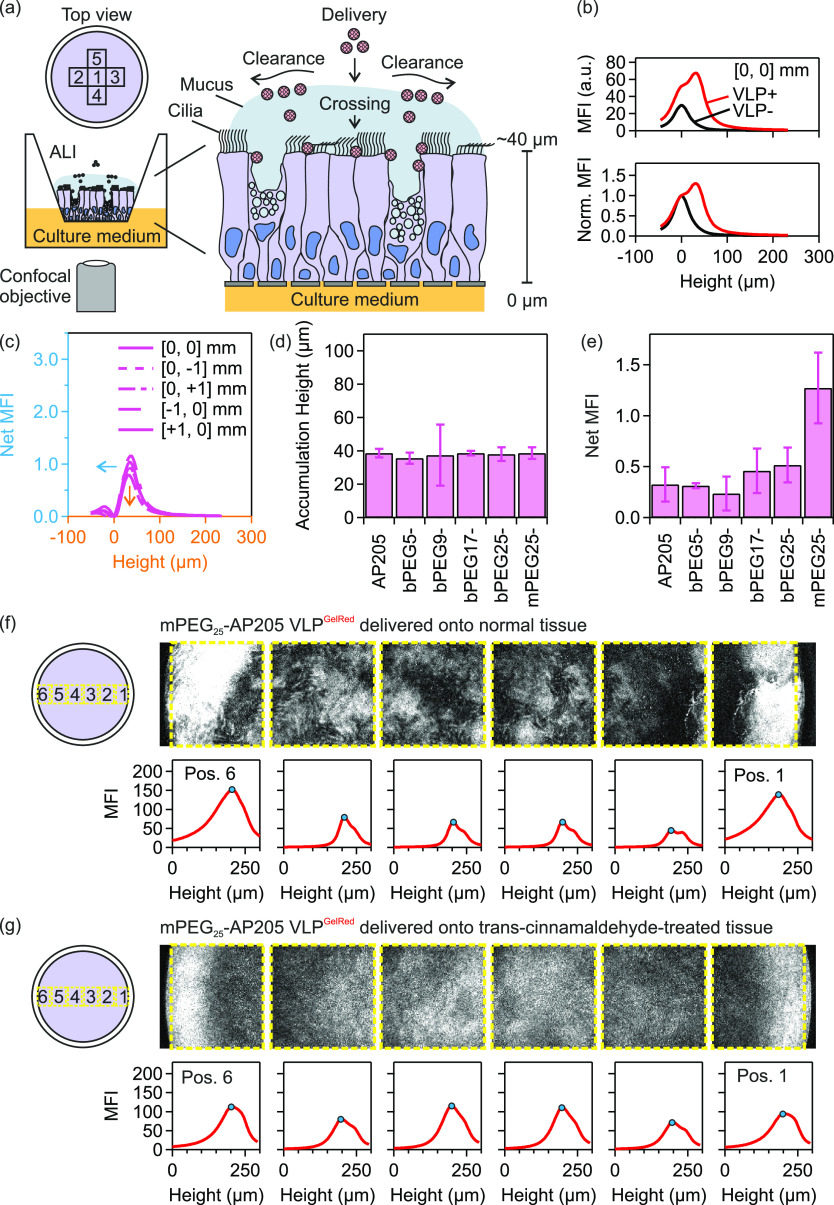
Translocation through mucus of AP205 VLPs in *in vitro* 3D human nasal epithelial tissues with mucociliary clearance. (a) Schematic of *in vitro* nasal epithelial tissue and delivery of fluorescently labeled virus-like particles (VLPs) from the apical side. Using laser scanning confocal microscopy (objective lens 10×, N.A. 0.3), the lateral distribution and vertical translocation through mucus of VLPs were monitored at several position 1: [0, 0] mm, 2: [−1, 0] mm, 3: [+1, 0] mm, 4: [0, −1] mm and 5: [0, +1] mm. (b) Mean fluorescence intensity (MFI) and normalized MFI (norm. MFI) variation with height collected in the center of tissue 3. MFI and norm. MFI spectra prior to VLP delivery, VLP–, and after the delivery of mPEG_25_-AP205 VLP^GelRed^, VLP+, are shown. The height of semipermeable membrane is set to 0 μm. (c) Subtracted (or net) MFI spectra on five positions showing the height location of VLP accumulation and the net MFI. (d, e) The accumulation height (d) and the net MFI corresponding to the translocated amount of VLPs (e) of native and PEGylated VLPs on tissue 3. (f) Delivery of mPEG_25_-AP205 VLP^GelRed^ to tissue 3. (g) Delivery of mPEG_25_-AP205 VLP^GelRed^ to trans-cinnamaldehyde-treated tissue 3. The yellow box denotes the area of mean fluorescence intensity (MFI) calculation and blue circle the height location of VLP accumulation. In these figures, height starts from above the air–liquid interface (ALI) and increases in the direction of cell layer to below the semipermeable membrane. The height of the semipermeable membrane is approximately 250 μm. The individual confocal images on lateral positions 1 to 6 were taken at a fixed height distance from the semipermeable membrane.

The schematic of the experiments is shown in Figure 5(a). On delivery of fluorescently labeled VLPs from the apical side, the VLPs translocated through mucus by diffusion and simultaneously transported in flowing mucus in the direction of mucociliary clearance to edges of the tissue. The lateral distribution and vertical translocation were monitored using laser scanning confocal microscopy (LSCM).

In Figure 5(b), representative examples of mean fluorescence intensity (MFI) variation with height collected in the center, [0, 0] mm, prior to VLP delivery (VLP−) and after the delivery of mPEG_25_-AP205 VLP^GelRed^ (VLP+) are shown. In the former case, the location of maximum MFI was at the height level of the semipermeable membrane which was assigned to 0 μm. After VLP delivery, the maximum of MFI shifted to a higher height value (about 40 μm) while the peak associated with semipermeable membrane was observable. The MFI vs height spectra were normalized (norm. MFI) by the MFI values at height = 0 μm, Figure 5(b). The normalized MFI spectrum of VLP- was then subtracted from that of VLP+ to give the net MFI spectrum. The net MFI spectra from the center, [0, 0] mm, together with spectra at positions [−1, 0] mm, [+1, 0] mm, [0, −1] mm, and [0, +1] mm, are shown in Figure 5(c). These spectra provided information about the height location of VLP accumulation after delivery and the net value of MFI at the accumulation heights, which corresponded to the translocated amount of VLPs. In all experiments, it was ensured via bright field microscopy that the cilia were beating before and after VLP delivery using established procedures.^60^

A typical accumulation height and net MFI (corresponding to translocated amount) of native and PEG-cross-linked VLPs are shown in Figure 5(d) and (e). A similar accumulation height between these VLPs was expected as tissues from the same donor were used (tissue 3, cf. S8). Furthermore, as shown in Figure 5(e), similar translocated amounts between the native and PEG-cross-linked VLPs were obtained. This observation indicated that PEG-cross-linking did not restrict nor increased VLP translocation through mucus. These results were consistent on other tissues (Figure S8–1).

We then investigated the translocation behavior of PEGylated but not cross-linked VLP, namely mPEG_25_-AP205 VLP^GelRed^. We found that mPEG_25_-AP205 VLP^GelRed^ showed a similar accumulation height (Figure 5(d)), however it had an increased net MFI (translocation amount) which exceeded native VLP by two folds (Figure 5(e)). Particularly as compared to bPEG_25_-AP205 VLP, this result indicated a higher mucus permeability of the mPEG_25_-AP205 VLP construct. Consistent with previous reports,^61−63^ a potential explanation is an effective steric hindrance interactions with mucin glycoproteins from one-end conjugated PEGs.

Particularly in the airways, the mucus is subject to mucociliary clearance which potentially reduces VLP translocation efficiency by pushing the mucus out of the airways.^58^ In *in vitro* 3D human nasal epithelial tissues, mucociliary clearance has been measured to transport mucus at a speed of about 40–200 μm/s (measured at 1–3 mm from the tissue center^64,65^) in a swirling rotation to the edges of the tissue (Figure 5).

We therefore investigated the effect of outward-swirling mucociliary clearance on the translocation and accumulation of VLPs.^64^ mPEG_25_-AP205 VLP^GelRed^ was delivered in normal tissue condition or when the tissue was treated with trans-cinnamaldehyde. Analysis of the lateral distribution of accumulated VLPs across the tissue (positions 1 to 6) under normal conditions (Figure 5(f)) showed that a noticeable portion of VLP was cleared to the edge of the plastic insert in flowing mucus while a portion translocated similar to the observations already discussed. However, the lateral distribution of accumulated VLPs was more uniform across the tissue when ciliary beating was stopped (Figure 5(g)). Additional examples of the accumulated VLP distribution when ciliary beating was inhibited are shown in Figure S8–2.

### Compatibility of Surface Cross-Linking with Vaccine Function of VLPs

A major application of VLPs is in mucosal delivery of vaccine antigens. We therefore investigated if surface cross-linking may interfere with this function by measuring antibody induction against AP205 coat protein in native and PEG-cross-linked VLPs. Subcutaneous vaccination model using C57BL/6J specific pathogen free (SPF) mice was selected as a setting where vaccine stability is thought not to be a major limitation on performance. Accordingly, subcutaneous injections of VLPs without additional adjuvant were performed on Day 0 and 10. The mice were sacrificed on Day 20 and anti-AP205 serum IgG titers detected using enzyme-linked immunosorbent assay (ELISA).

**Figure 6:**
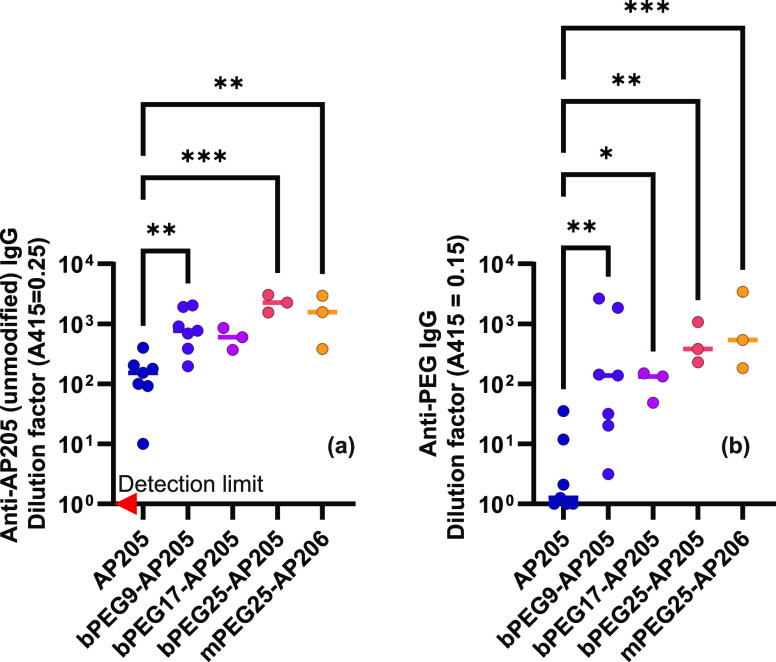
Immune activation by PEG-cross-linked AP205 VLPs. C57BL/6J SPF mice were subcutaneously injected with native (*n* = 7), PEG-cross-linked (*n* = 13), or PEGylated but not cross-linked (*n* = 3) AP205 VLPs on Day 0 and Day 10 and sacrificed on Day 20. Serum antibody (IgG) titers against (a) AP205 coat protein and (b) PEG was measured by ELISA. Statistical significance was determined by ordinary one-way ANOVA with Dunnett’s multiple comparisons test, with a single pooled variance on log-normalized data (*P = 0.0332; **P = 0.0021; ***P = 0.0002). Red arrow in (a) indicates detection limit.

The serum antibody titers against AP205 coat protein are shown in Figure 6(a). As expected, native VLP was immunogenic, and we easily detected specific serum IgG antibodies against AP205 coat protein.^40,41^ Surprisingly, PEG-cross-linking, nor simple PEGylation of VLP did not shield immune activation against the coat protein; rather, specific antibody induction was increased. Indeed, given our results, it is not likely that PEGylation is shielding important AP205 epitopes, as may be the case for other PEGylation regimens that decrease antigenicity.^66^ Rather we could hypothesize that early priming of PEG-specific antibodies results in improved antigen-presentation upon boosting or PEGylation alters the half-life of antigen in the lymph nodes.^17−25^ Potentially, other parameters such as VLP stiffness could also be involved (i.e., the higher stiffness of PEGylated VLPs compared to native VLP, cf. Figure 2 and Figure 3). We note that the effect is mild, which would make further mechanistic dissection challenging.

Antibody induction against the PEG coating was also investigated. Consistent with the strong adjuvanticity of VLPs,^67^ the anti-PEG ELISA in Figure 6(b) showed that the injection of PEGylated VLPs, compared to the negative control, i.e., native VLP, resulted in anti-PEG IgG induction.

## Conclusions

VLPs have similar physical characteristics to their viruses of origin, including size, proteinaceous core–shell structure, and patchy surface charge distribution, and like viruses are prone to aggregation, disintegration, and sticking to surfaces rendering them nonfunctional.^68^ Enhancing the stability and survival of VLPs is therefore justified in future developments of VLP nanobiotechnology, particularly for mucosal applications. We investigated surface cross-linking as a means to achieve the latter. We found that it increased the colloidal stability of VLPs at low pH as it protected them against proteases. Susceptibility to mechanical agitation was introduced as another limiting factor to VLP mucosal application. Therefore, it was also logical to mechanically reinforce VLPs and surface-cross-linking was found to increase the stiffness and strength of AP205 VLP. Mucosal applications of VLPs are faced with other physiological challenges, namely translocation through a thick mucus layer. However it seems that mucus is a poor barrier to VLPs of a size below about 100 nm, including AP205 VLP (27.6 nm), and VLPs of human papilloma virus and Norwalk virus (respectively, 55 and 38 nm^69^). Considering viruses that infect mucosal surfaces, including polio (28 nm), hepatitis B (43 nm), adenoviruses (60–90 nm), rotavirus (75 nm), human immunodeficiency virus (120 nm), and herpes simplex virus (180 nm),^70^ one may conclude that small viruses are capable of deeply penetrating the mucus. This is expected when the pore size of mucus gel (≥50 nm) exceeds the size of these viruses. However, penetration is further facilitated by the patchy surface charge distribution on capsids.^69,70^ Therefore, structural point mutations as well as macromolecule entanglement with mucin glycoproteins can potentially alter the diffusion of VLPs in mucus and ought to be investigated prior to application.^71^

Finally, our results suggest that the material properties of VLPs are relevant variables that should be further investigated to improve mucosal applications. As schematically shown in Figure 1(a), both chemical, enzymatic, and physical stresses can affect the efficacy of VLP delivery to the respiratory or intestinal mucosal epithelium. In this work, cross-linking of multiple surface sites was shown to be a promising approach to improve and extend the range of VLP mucosal functions by increasing resistance to degradation and improving physical stability. These modifications actually slightly enhance even parenteral vaccination indicating that the tethers did not shield the coat proteins from immune recognition, and that increased biochemical stability may also be beneficial systemically. PEG immunogenicity is currently an unwanted side-effect that may be overcome with use of alternative cross-linkers in the future. Critically, our work demonstrates the potential to improve the current VLP system to generate structures compatible with overall enhanced functionality, strength, stability, and immunogenicity at mucosal surfaces.

## Materials and Methods

### Experimental Design

Surface cross-linking of virus-like particle (VLP) capsids using polyethylene glycol (PEG) tethers was investigated against physiological challenges to assess its performance on mucosal surfaces. The model native VLP was derived from the coat protein of Acinetobacter phage, namely AP205 VLP. PEG tethers included two-end functional PEG molecules of various molecular weights. The PEG-cross-linked AP205 VLPs were initially evaluated in terms of the reaction products and the sites of PEG conjugation to reactive surface sites (using a combination of dynamic light scattering, transmission electron microscopy, reducing SDS–polyacrylamide gel electrophoresis, electrospray ionization mass spectroscopy and nano ultraperformance liquid chromatography–MS) and then investigated for the aggregation behavior against pH (using dynamic light scattering), disintegration against digestive enzymes from pig and mouse gastric fluid (using agarose gel electrophoresis), resistance to mechanical force (using atomic force microscopy), and penetration in mucus in the presence of motile cilia and mucociliary clearance (using *in vitro* 3D human nasal tissues).

### Production of AP205 VLP

The biosynthesis of AP205 VLP is reported in details in our previous study.^37^ Briefly, it includes the expression of recombinant AP205 coat proteins in bacterial cytoplasm which then self-assemble into AP205 VLPs. After the expression of VLP, a series of steps consisting of bacterial lysis, digestion of RNA and DNA contaminants, and extraction of lipids were performed prior to precipitation and dialysis against PBS to produce purified AP205 VLPs. Plasmids were generated by custom DNA synthesis (Twist Bioscience, San Francisco, CA, USA) of the expression cassettes and cloning into the pRSFDuet-1 (Novagen) backbone between the PfoI and BshTI restriction sites. Sequences of the plasmids are available via the ETH-Zürich research collection at DOI: 10.3929/ethz-b-000556182. The sequence of AP205 monomer and the description of plasmid are reported in S1.

### PEGylation of AP205 VLP

The functional group on the extremities of homobifunctional PEG cross-linker molecules (bPEG_*n*_, *n* = 5 to 25) and on one extremity of monofunctional PEG molecules (mPEG_*n*_, *n* = 12 and 25) was NHS-ester which is reactive to primary amine of lysine residues and N-terminus of AP205 coat proteins. Reactions with native AP205 VLP proceeded at 1–100x molar ratio of cross-linker molecule to AP205 monomer (molar ratio = [functional moleucle]/[AP205 CP]). The PEG molecule was dissolved in anhydrous and molecular sieves dried dimethyl sulfoxide (DMSO, Sigma-Aldrich) to a concentration of 100 mM. An appropriate amount was then added to AP205 VLP suspension at 4 mg/mL in PBS. The reaction was left for 24 h at 4 °C. Then after, it was quenched by adding Tris·HCl (Trizma base, Sigma-Aldrich, and hydrochloric acid 37%, VWR) at 20–50 mM and incubating for 15 min. Unreacted PEG molecules and Tris were removed using Zeba Spin Desalting Column (ThermoFisher) with molecular weight cutoff at 7 kDa. Other reactive molecules including BS^3^ and Sulfo-NHS were used similarly; however, they were initially dissolved to 100 mM in PBS. A summary of the physical properties of these molecules is provided in S1.

### Fluorescent Staining of VLPs

We defined a mixing ratio *x* = mass of stain/mass of VLP = 10^–2^, and a final stained VLP concentration equal to 5.3 mg/mL. (To calculate the mass of stain, we assumed 10,000x to correspond to 10 mg/mL.) GelRed (10,000× in water, biotium) or SYBRGold (10,000× in DMSO) were used and staining proceeded for 30 min. To remove unbound dye, the solution of stained VLP was buffer exchanged with pure PBS using Zeba Spin Desalting Columns (7,000 MWCO, ThermoFisher). We note that since the columns contained sodium azide, it was important to buffer the column with pure PBS prior to cleaning the stained VLP solution. The stained VLP solution was finally filtered using 0.2 μm centrifuge filters (Corning Costar Spin-X centrifuge tube filters) to remove possible aggregates. The intensity of stained VLPs was monitored over time using laser scanning confocal microscopy and showed no noticeable reduction over a course of at least two weeks.

### Transmission Electron Microscopy (TEM)

Four μL VLP solution (0.1 mg/mL) was deposited on glow-discharged (45 s, 25 mA, negative charge) carbon-coated grids (Ted Pella) for 1–5 min, after which the excess liquid was removed using filter paper. Then the grid was rinsed with 5 μL of PBS to remove loosely bound VLPs. Subsequently, 3 μL of 2% uranyl acetate was applied and immediately removed with filter paper prior to a second treatment with 3 μL of 2% uranyl acetate for 20 s. Excess liquid was again collected using filter paper and the grid was let dry at room temperature (RT). Data acquisition was performed using a Morgani transmission electron microscope operating at 100 kV acceleration voltage.

### Electrospray Ionization Mass Spectrometry (ESI-MS)

ESI-MS was acquired on a Synapt G2-Si quadrupole time-of-flight mass spectrometer (Waters, UK) with a capillary voltage of 3 V, a cone voltage of 50 V and a source temperature of 100 °C. VLPs at a concentration of 1 mg/mL were reduced by dithiothreitol (DTT) at a final concentration of 50 mM. The reduction was performed for 1 h at RT and at pH 8. The samples were acidified with 1% formic acid (Thermo, USA), desalted using C4 Zip Tips (Millipore, USA) and analyzed in methanol:2-propanol:0.2% formic acid (30:20:50). The solutions were infused through a fused silica capillary (ID 75 μm) at a flow rate of 1 μL/min and sprayed through Pico Tips (ID 30 μm). The last were obtained from New Objective (Woburn, MA, USA). Recorded *m*/*z* data were deconvoluted using the MaxEnt1 software (Waters, UK) with a resolution of the output mass of 0.5 Da per channel and a Uniform Gaussian Damage Model at the half-height of 0.5 Da.

### Nano Ultraperformance Liquid Chromatography–MS (nanoUPLC-MS/MS)

NanoUPLC-MS/MS was acquired on Thermo FUSION mass spectrometer (Thermo, Germany) coupled to a nano-M-class UPLC system (Waters). BPEG_9_-AP205 VLP at a concentration of 1 mg/mL was digested by incubating 3 μL of the sample with 42 μL of 25 mM ammonium bicarbonate (pH 8.5) and 1 μL of TCEP (100 mM) for 1 h at 37 °C. Reduced protein was incubated with 2 μL chloroacetamide (500 mM) for 1 h at 37 °C. Then after, 5 μL trypsin (0.1 μg/μL in 10 mM HCl) was added and the solution further incubated overnight at 37 °C. When needed the pH was adjusted to pH 8. At the end of this procedure, the sample was dried using SpeedVac. Digested and dried sample was dissolved in 20 μL ddH_2_O with 0.1% formic acid and diluted 5 times before transferring to autosampler vials. Peptides were resuspended in 2.5% acetonitrile with 0.1% formic acid and loaded onto a nanoEase M/Z Symmetry C18 (75 μm × 20 mm, 100 Å, 3 μm particle size) and separated on a nanoEase M/Z HSS C18 T3 (75 μm × 150 mm, 130 Å, 1.7 μm particle size) at a constant flow rate of 300 nL/min, with a column temperature of 50 °C and a linear gradient of 2–32% acetonitrile/0.1% formic acid in 79 min, and then 32–45% acetonitrile/0.1% formic acid in 10 min, followed by a sharp increase to 98% acetonitrile in 2 min and then held at 98% for another 10 min. Mass spectrometer was operated under data-dependent acquisition (DDA), one scan cycle comprised of a full scan MS survey spectrum, followed by up to 12 sequential higher-collisional energy (HCD) MS/MS on the most intense signals above a threshold of 1e4. Full-scan MS spectra (600–2000 *m*/*z*) were acquired in the FT-Orbitrap at a resolution of 70,000 at 400 *m*/*z*, while HCD MS/MS spectra were recorded in the FT-Orbitrap at a resolution of 35,000 at 400 *m*/*z*. HCD was performed with a target value of 1 × 10^5^ and normalization collision energy 25 NCE (normalize collisional energy) was applied. Autogain control (AGC) target values were 5 × 10^5^ for full Fourier transform (FT) MS. For all experiments, dynamic exclusion was used with a single repeat count, 15 s repeat duration, and 30 s exclusion duration. The acquired MS data were processed for identification using the Byonic search engine (PMI). The spectra were searched against AP205 VLP sequence and AP205 VLP sequence with *E. coli* database. The following modifications were included: (a) variable modifications, namely oxidation at N-terminus methionine and carbamidomethylation at cysteine (C), and (b) user-defined modifications, namely 478.2565 (C_22_O_11_H_38_) was set for PEG_9_-cross-linked peptides and 496.2565 (C_22_O_12_H_40_) for one-end PEG_9_ conjugation at a lysine residue or N-Terminus. In this analysis, the raw data were analyzed by two software, PMI Byonic search engine and SIM 1.5.5.3 (Spectrum identification machine).^72^ In addition, two FastA databases with and without starting methionine were used to confirm the N-terminus.

### SDS–Polyacrylamide Gel Electrophoresis (SDS-PAGE)

Denaturing and reducing SDS-PAGE measurements were carried out using Criterion XT Precast Gel 12% Bris-Tris. Samples of a few μg of native and PEG-modified VLPs were denatured and reduced in Laemmli buffer in the presence of 1 M Tris(2-carboxyethyl) phosphine hydrochloride (TCEP·HCl) at 80 °C for 20 min prior to electrophoresis. Proteins were visualized by Coomassie staining.

### Dynamic Light Scattering (DLS)

DLS was performed at a fixed angle of 173° by averaging 3 runs of 30-s long each using Zetasizer Nano (Malvern Panalytical). The time correlation function of scattered intensity was analyzed by the cumulant as well as CONTIN methods. The VLP concentration was initially adjusted to 0.1 mg/mL in filtered PBS prior to measurements.

### Gastric Fluid Collection

Pig gastric content was collected from the stomach of conventionally reared, outbred pigs with different genetic contributions of French-Swiss Landrace and Swiss Large White. Total gastric content was collected. Mouse gastric content was obtained by collecting total stomach content from several specific opportunistic pathogen-free (SPF) C57BL/6J WT mice. The gastric content from several mice was pooled to have sufficient volume. Pig or mouse gastric content was centrifuged for 5 min at 16000 rpm to separate the nondigested food particles. After the collection of the supernatant (gastric fluid), it was filtered using 0.22 μm sterile filter and the pH measured using a laboratory pH-meter.

### Agarose Gel Electrophoresis

AP205 is a single-stranded RNA bacteriophage. During recombinant expression of the AP205 VLP in *E. coli*, the capsid packages free RNA from the bacterial cytoplasm. Ethidium bromide (EB) can diffuse inside the capsid through structural pores and stain the nucleic acids. We have therefore used EB during agarose gel electrophoresis. On the gel, the nucleic acid bands migrated approximately related to the total VLP size and charge. Loss of VLP integrity (e.g., due to digestion when exposed to gastric fluid) resulted in the loss of the packaged nucleic acid and therefore the loss of EB signal on agarose gel. Using this methodology, two sets of measurements were performed after gastric digestion of the VLPs. (A) VLPs of about 1 mg/mL were diluted 1:10 in filtered (0.22 μm) pig or mouse gastric fluid and incubated for 5 min, 30 min, 1 h and 1.5 h or 2 h at 37 °C. After each incubation, the samples were placed on ice. Native agarose gel electrophoresis of PBS- or gastric fluid-treated VLPs was carried out in 0.8% agarose in TAE buffer (40 mM Tris, 0.1% acetic acid, 1 mM EDTA) with 0.5 μg/ml of ethidium bromide for visualization. Samples of 5 μg VLP were electrophoresed at 90 V for 90 min, then imaged on a UV transilluminator. The measurements are shown in Figure 1(e) and in Figure S7–1 to S7–3. (B) For semiquantification of the degradation, VLPs of about 1 mg/mL were diluted 1:4 in pig gastric fluid and incubated for 1 min to 2 h at 37 °C while shaking at 300 RPM. After each incubation, an aliquot was added to a solution of cOmplete, EDTA-free Protease Inhibitor Cocktail 2× (Sigma-Aldrich) and DNA Loading Dye 1× (ThermoFisher), then placed on dry ice. Native agarose gel electrophoresis was similar to (A). The measurement is shown in Figure S7–4.

### Atomic Force Microscopy (AFM)

The schematic of AFM measurements is shown in Figure 2(a). After an initial calibration, which also included the calibration of PeakForce tapping mode, on freshly cleaved mica, the tip was brought in proximity to VLPs immobilized at a concentration of 5 μg/mL on Highly Oriented Pyrolytic Graphite (HOPG, Ted Pella, Inc.) The calibrated parameters included stiffness (59.4–82.0 pN/nm) using thermal method and optical lever sensitivity using cantilever–mica contact region,^73^ and drive3 amplitude sensitivity. During the transport of cantilever from mica to HOPG, the cantilever remained wet, and the laser spot was checked to remain on the same spot. The calibration values were remeasured on mica at the end of the measurements to detect any noticeable variation to the calibrated parameters. Single VLPs were localized on HOPG by imaging in PeakForce tapping mode (peak force 50 pN, tapping frequency 1 kHz, amplitude 30 nm). Generally, images of individual VLPs were acquired at a scan rate of 0.7 Hz with 200 × 200 or 500 × 500 nm^2^ scan size and a resolution of 64 points per line, leading to a pixel size of 3 nm. After localization, we used “Point & Shoot” function (NanoScope, Bruker) to place the AFM tip on the apex of selected VLPs before applying indentation using Ramp mode. The VLPs were indented up to a threshold force value of 400 pN at a preset frequency of 1 Hz, which resulted in an indentation force rate of about (4–9)×10^3^ pN/s. Indented particles were imaged again in PeakForce tapping to detect signs of degradation, drift, or displacement due to tip lateral forces. The height of individual VLPs before (*H*_*i*_) and after (*H*_*f*_) indentation were calculated by placing a trace line over the particles on images previously flattened using Gwyddion 2.47.^74^ For stiffness evaluation, we proceeded as follows: deflection versus piezo displacement was converted to force versus tip separation using protocols written in Igor Pro (Wavemetrics). Fit to force versus indentation distance (*F* – δ) curve in the linear response part of the tip–VLP contact region gave the stiffness (*k* = *dF*/*dδ*) and the indentation (δ = Δ*F*/*k*). The fit was performed in the force range 50 pN ≤ *F* ≤ 250 pN, and only those VLPs with indentation range 2.0 ≤ δ ≤ 6.0 nm were selected for averaging. Only one indentation per VLP was used for the evaluations. The indentation force rate (*Ḟ*) was calculated from a linear fit to the force versus indentation time (*F* – *t*) curve in the same region were the stiffness of VLP was calculated. Strain rate was then calculated from the relation *γ̇* = *Ḟ*/*kδ*. Average stiffness was calculated from at least duplicate independent VLP preparations. UV-ozone (Novascan) cleaned AC40 BioLever Mini cantilevers (Bruker) were used. Dimension FastScan (Bruker) was used for imaging and force spectroscopy.

### Small Angle X-ray Scattering (SAXS)

We used a Xeuss 2.0 (Xenocs, France) instrument with a microfocused X-ray source at the Laboratory Léon Brillouin (LLB NIMBE CEA Saclay). The Cu Kα radiation (λ_Cu Kα_ = 1.5418 Å), and the data were collected by a 2D Pilatus 1 M detector (Dectris, Switzerland). The scattering vector *q* = 4π/λsin(θ/2), with θ being the scattering angle was calibrated using silver behenate. Data were collected and azimuthally averaged to produce unidimensional intensity versus *q*, with *q* interval of 0.005 to 0.1 Å^–1^. The measurements were performed at 25 °C. The scattering intensity was collected for 2600 s. The intensity was corrected for sample thickness, transmission, and acquisition time. The modeling of the integrated data was performed with a core–shell form factor, and a hard sphere structure factor, when needed, using the SasView software.

### *In Vitro* 3D Human Nasal Epithelial Tissue

*In vitro* human nasal epithelial tissues from single donors (MucilAir) were obtained from Epithelix (Switzerland). The tissues were maintained according to company’s protocols and included tissue 1 from a Caucasian 64-year-old female subject (MD0860), tissue 2 from a Caucasian 46-year-old male subject (MD0871), and tissue 3 from a Caucasian 38-year-old male subject (MD0774). The subjects were nonsmokers and had no history of respiratory pathology.

### Inhibition of Mucociliary Clearance

Ciliary beating was inhibited using trans-cinnamaldehyde (97%, C80687, Sigma-Aldrich). Trans-cinnamaldehyde was dissolved in MucilAir culture medium to a concentration of 30 mM. To improve solubility, freshly prepared solution was sonicated for 15 min and the solution was kept at 37 °C prior to filling the basal channel of MucilAir monodonor nasal epithelial tissue. After 20 min of incubation, the ciliary beating stopped, and no recovery was observed for up to 2 h.^75^

### Delivery of VLPs to *In Vitro* 3D Human Nasal Epithelial Tissue

Tissues were incubated in a humidified-CO_2_ chamber at 37 °C. After a period of equilibration 0.5–1 h, 5 μL of VLP solution (5.3 mg/mL) was delivered from the apical side. The delivery was onto the center of the tissue.

### Laser Scanning Confocal Microscopy (LSCM)

Lateral distribution and vertical translocation of fluorescently labeled VLPs through mucus were then monitored using laser scanning confocal microscopy (LSCM, Leica SP5 confocal microscope). We used a 10× objective lens with N.A. 0.3. Excitation wavelength λ_ex_ = 488 nm was used for SYBRGold-stained VLPs and Salmonella vaccine with detection wavelength λ_em_ = 500–590 nm. λ_ex_ = 514 nm was used for GelRed-stained VLPs with λ_em_ = 550–650 nm. The channel spectral detection was set to HyD (Gain equal to 100). The mean fluorescence intensity (MFI) variation with height was collected in the center [0, 0] mm, and on positions [−1, 0] mm, [+1, 0] mm, [0, −1] mm and [0, +1] mm prior to, during, and after VLP delivery. Leica LAS AF software was used for saving the images. Analysis was performed using ImageJ.

### Mice

All animal experiments were performed in accordance with Swiss Federal regulations approved by the Commission for Animal Experimentation of the Kanton Zurich (license ZH009/2021, Kantonales Veterinäramt Zürich, Switzerland). SPF C57BL/6J WT mice were used in all experiments. Mice were bred and housed in individually ventilated cages with a 12 h light/dark cycle in the ETH Phenomics Center (EPIC, RCHCI), ETH Zürich, and were fed a standard chow diet. All mice included in experiments were 7 weeks or older and objectively healthy as determined by routine health checks. Wherever possible an equal number of males and females was used in each experimental group.

### Subcutaneous Injection

50 μg of VLP in 100 μL of PBS was subcutaneously injected into the loose skin over the interscapular area of C57BL/6J SPF mice on Day 0 and Day 10. The mice were euthanized on Day 20. Blood was collected by cardiac puncture, and serum separated by spinning 1.1 mL serum gel tubes (Sarstedt) at 10,000*g* for 5 min at 20 °C in a conventional table-top centrifuge, and heat-inactivated for 30 min at 56 °C.

### Enzyme-Linked Immunosorbent Assay (ELISA) for Detection of Anti-AP205 IgG Response

50 μL of AP205 VLP solution (5 μg/ml) was pipetted into an appropriate number of wells of ELISA plates (4 Nunc MaxiSorp flat bottom 96-well plates) and the VLP allowed to adsorb overnight at 4 °C. The wells were washed three times with a wash buffer (0.05% Tween 20 in PBS) and blotted. The wells were blocked using 150 μL of blocking buffer (2% bovine serum albumin in PBS) for 1.5 h at RT. Subsequently, the wells were washed five times with the wash buffer and blotted. Mouse sera were diluted ten times in blocking buffer, and sequentially in 1:3 dilution steps added to the wells of ELISA plates. The plates were incubated for 2 h at RT to allow the binding of serum IgG to AP205 proteins. Goat antimouse IgG-HRP (Sigma) was diluted a thousand-fold in blocking buffer, and 50 μL of solution added to the wells and incubated for 1 h at RT. The wells were washed five times using the wash buffer and blotted. Finally, 150 μL of freshly prepared substrate solution (consisting of 0.1 M NaH_2_PO_4_ (substrate buffer), 2,2′-azino-bis(3-ethylbenzothiazoline-6-sulfonic acid (1–2 mg per 10 mL of substrate buffer), and H_2_O_2_ (10 μL per 10 mL)) was added to each well. The plate was incubated in the dark for 40 min. On Tecan (Tecan Infinite 200 PRO microplate reader), absorbance was measured at 415 nm. The reference wavelength for the subtraction of background signal was set to 480 nm.

### Enzyme-Linked Immunosorbent Assay (ELISA) for Detection of Anti-PEG IgG Response

Mouse sera were diluted ten times in PEG dilution buffer (PEGD50–1, Life Diagnostics, Inc.), and sequentially in 1:3 dilution steps added to the wells of PEG-BSA coated ELISA plates (PBSA20PL, Life Diagnostics, Inc.) The plates were incubated for 2 h at RT to allow antibody binding. The wells were washed five times with 300 μL PEG wash buffer (PEGW50–20, Life Diagnostics, Inc.) and blotted. Goat antimouse IgG-HRP (AP124P, Sigma-Aldrich) was diluted a thousand-fold in PEG dilution buffer and 100 μL of this solution was added to each well with an incubation of 1 h at RT. The wells were washed and blotted again. Finally, 150 μL of freshly prepared substrate solution was added to each well. The plates were incubated in the dark for 40 min. On Tecan (Tecan Infinite 200 PRO microplate reader), absorbance was measured at 415 nm. The reference wavelength for the subtraction of background signal was set to 480 nm.

### Statistical Analysis

AFM and LSCM results were expressed as mean ± standard deviation analyzed by Igor Pro from WaveMetrics. *In vivo* mouse vaccination results were assessed using ordinary one-way ANOVA with Dunnett’s multiple comparisons test using GraphPad Prism (*P = 0.0332; **P = 0.0021; ***P = 0.0002).

## Data Availability

All data used in the analyses are available without limitation from the first author.

## Supporting Information Available

AP205 coat protein sequence and the molecules used to modify AP205 VLP (S1); Hydrodynamic diameter of bPEG_9_-AP205 VLP at varying molar ratio of BS(PEG)_9_ to AP205 monomer (Figure S2–1); hydrodynamic diameter of bPEG_25_-AP205 VLP at varying molar ratio of BS(PEG)_25_ to AP205 monomer (Figure S2–2); TEM images of native AP205 VLP, and bPEG_9_-AP205 VLP, bPEG_13_-AP205 VLP, bPEG_17_-AP205 VLP, bPEG_21_-AP205 VLP, bPEG_25_-AP205 VLP (Figure S3–1); reducing SDS-PAGE of native AP205 VLP, AP205 VLP in mock reaction, PEG-cross-linked, and PEGylated but not cross-linked AP205 VLPs together with densitometry profiles (Figure S4–1); additional reducing SDS-PAGE gels (Figure S4–2 and Figure S4–3); ESI-MS data of AP205 VLP, bPEG_9_-AP205 VLP, bPEG_13_-AP205 VLP, bPEG_17_-AP205 VLP, bPEG_21_-AP205 VLP and bPEG_25_-AP205 VLP (Figure S5–1 to Figure S5–6); hydrodynamic diameter of AP205 VLP, bPEG_9_-AP205 VLP and bPEG_21_-AP205 VLP at varying pH (Figure S6–1 and Figure S6–2); enzymatic stability of native and PEGylated AP205 VLPs in pig and mouse gastric fluids (Figure S7–1 to Figure S7–4); translocation of native and PEGylated AP205 VLPs through mucus in *in vitro* 3D human nasal epithelial tissues (Figure S8–1 and Figure S8–2) (PDF)
