# Supplementary material for: Surface Cross-Linking by Macromolecular Tethers Enhances Virus-like Particles’ Resilience to Mucosal Stress Factors
Source: ACS Nano. 2024 Jan 18;18(4):3382–96. doi: 10.1021/acsnano.3c10339 (PMC10832050; doi:10.1021/acsnano.3c10339)
Supplement: Supplementary file 1 — nn3c10339_si_001.pdf [file nn3c10339_si_001.pdf]

Surface Crosslinking by Macromolecular Tethers Enhances Virus-Like Particles Resilience to Mucosal Stress Factors

Supplementary Information

Ahmed Ali [a], Suwannee Ganguillet [a], Yagmur Turgay [a], Timothy G Keys [a], Erika Causa [b], Ricardo Fradique [b], Viviane Lutz-Bueno [c,d], Serge Chesnov [e], Chia-Wei Tan-Lin [e], Verena Lentsch [a], Jurij Kotar [b], Pietro Cicuta [b], Raffaele Mezzenga [a], Emma Slack [a,*], Milad Radiom [a,b,*]

[a] Department of Health Sciences and Technology, ETH Zürich, 8092 Zürich, Switzerland
[b] Biological and Soft Systems, Cavendish Laboratory, University of Cambridge, Cambridge CB3 0HE, U.K.
[c] Paul Scherrer Institute (PSI), Villigen 5232, Switzerland
[d] Laboratoire Léon Brillouin, CEA-CNRS (UMR-12), CEA Saclay, Université Paris-Saclay, Gif-sur-Yvette Cedex 91191, France
[e] Functional Genomics Centre Zürich (FGCZ), University of Zürich/ETH Zürich, Zürich 8057, Switzerland

* Emma Slack (emma.slack@hest.ethz.ch); Milad Radiom (milad.radiom@hest.ethz.ch)

Table of Contents

S1. AP205 coat protein sequence and the molecules used to modify AP205 VLP 4

AP205 sequence 4

Molecules used to modify AP205 VLP 4

S2. PEG-crosslinking at varying reaction molar ratios 5

$bPEG_9$-AP205 VLP 5

$bPEG_{25}$-AP205 VLP 6

S3. Morphology of PEG-crosslinked AP205 VLPs 8

S4. Characterization of PEG-crosslinked AP205 VLPs from the analysis of reducing SDS-PAGE 9

S5. Mass spectrometric characterization of PEG-crosslinked AP205 VLPs 12

Electrospray ionization mass spectroscopy (ESI-MS) – "AP205 monomeric region" 12

ESI-MS of AP205 VLP 12

ESI-MS of PEG-crosslinked AP205 VLPs 12

ESI-MS of $bPEG_9$-AP205 VLP 14

ESI-MS of $bPEG_{13}$-AP205 VLP 15

ESI-MS of $bPEG_{17}$-AP205 VLP 16

ESI-MS of $bPEG_{21}$-AP205 VLP 17

ESI-MS of $bPEG_{25}$-AP205 VLP 18

Nano ultra-performance liquid chromatography coupled to mass spectroscopy (NanoUPLC-MS/MS) analysis of $bPEG_9$-AP205 VLP – "AP205 dimeric region" 19

S6. Investigation of colloidal stability 22

Native AP205 VLP 23

Comparison of colloidal stability between native and PEG-crosslinked AP205 VLPs 24

S7. Investigation of enzymatic stability in gastric fluid 25

S8. Translocation of native and PEGylated AP205 VLPs through mucus in *in vitro* 3D human nasal epithelial tissues 29

Results of VLP delivery to tissues 1 and 2 29

Effect of mucociliary clearance on inhibition on lateral distribution and vertical translocation of VLPs 30

References.. 32

S1. AP205 coat protein sequence and the molecules used to modify AP205 VLP

AP205 sequence

MANKPMQPITSTANKIVWSDPTRLSTTFSASLLRQRVKVGIAELNNVSGQYVSVYKRPAPKPEG
CADACVIMPNENQSIRTVISGSAENLATLKAEWETHKRNVDTLFASGNAGLGFLDPTAAIVSSDT
TAGSGGAHATANATAHHHHHH

The plasmid used to produce AP205 monomers in *E. coli* cytoplasm is reported in the table below.

Plasmid	Description	Source
pTK190	Expression construct for AP205 VLP. pRSFDuet backbone with a custom expression cassette consisting of a T5 promoter with dual lac operators, RBS, open reading frame encoding the AP205 coat protein C-terminally fused to a 6xHis tag, followed by a T7 terminator.	This study

Molecules used to modify AP205 VLP

Molecule<br>Company, product code	Homobifunctional/ monofunctional	Spacer length (nm)	Molecular weight (Da)	Name of conjugated AP205 VLP
BS^3<br>bis(sulfosuccinimidyl)suberate<br>Thermo Scientific 21580	Homobifunctional	1.1	572.4	BS^3-AP205
$BS(PEG)_5$<br>Bis(succinimidyl) penta(ethylene glycol)<br>Thermo Scientific 21581	Homobifunctional	2.2	532.5	$bPEG_5$-AP205
$BS(PEG)_9$<br>Bis(succinimidyl) nona(ethylene glycol)<br>Thermo Scientific 21582	Homobifunctional	3.6	708.7	$bPEG_9$-AP205
$BS(PEG)_{13}$<br>Bis-$dPEG_{13}$-NHS ester<br>Quanta Biodesign 10954	Homobifunctional	5.0	884.9	$bPEG_{13}$-AP205
$BS(PEG)_{17}$<br>Bis-$dPEG_{17}$-NHS ester<br>Quanta Biodesign 10979	Homobifunctional	6.4	1061.1	$bPEG_{17}$-AP205
$BS(PEG)_{21}$<br>Bis-$dPEG_{21}$-NHS ester<br>Quanta Biodesign 10956	Homobifunctional	7.9	1237.3	$bPEG_{21}$-AP205
$BS(PEG)_{25}$<br>Bis-$dPEG_{25}$-NHS ester<br>Quanta Biodesign 10968	Homobifunctional	9.3	1413.6	$bPEG_{25}$-AP205
Sulfo-NHS<br>N-hydroxysulfosuccinimide<br>Thermo Scientific 24510	Monofunctional	0	217.1	Sulfo-NHS-AP205
m-$dPEG_{12}$-NHS ester<br>Quanta Biodesign 10262	Monofunctional	4.5	685.8	$mPEG_{12}$-AP205
m-$dPEG_{25}$-NHS ester<br>Quanta Biodesign: 11291	Monofunctional	9.0	1258.4	$mPEG_{25}$-AP205

S2. PEG-crosslinking at varying reaction molar ratios

Experiments using dynamic light scattering (DLS) aided in identifying a mixing ratio range that inhibited VLP aggregation. To prevent a large excess of free homobifunctional PEG molecules, all reactions were carried out at 10x molar ratio of crosslinker molecule to AP205 monomer.

bPEG$_9$-AP205 VLP

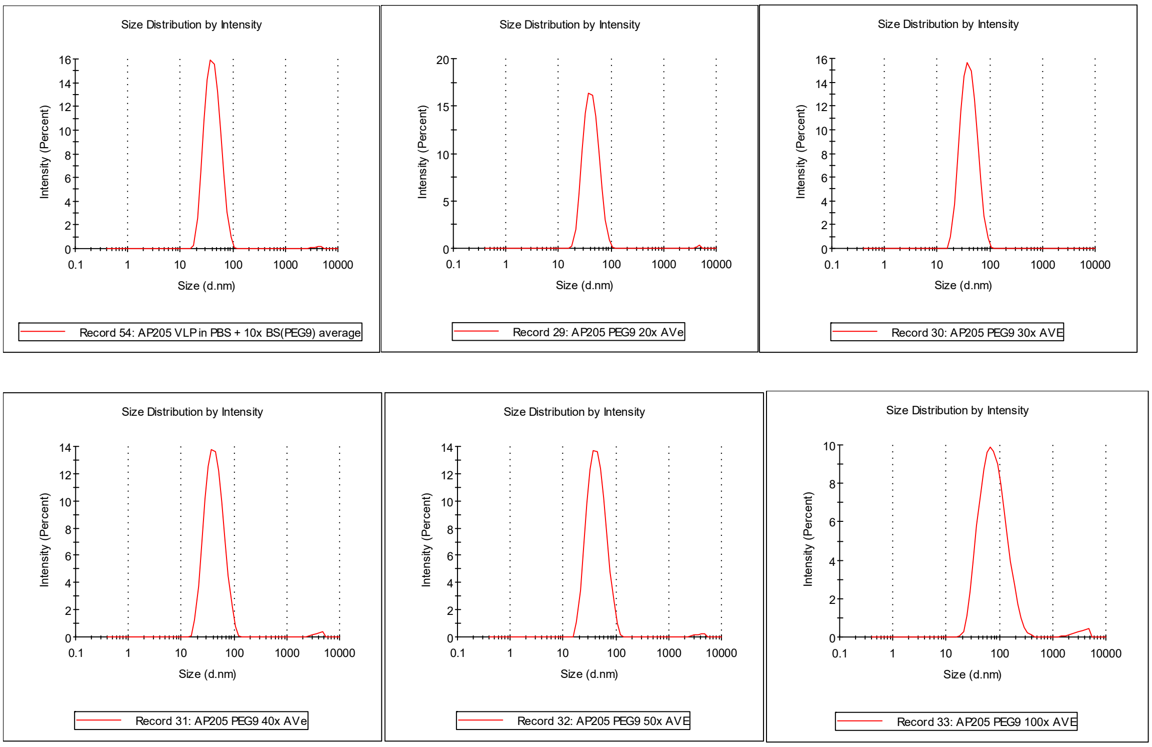

Figure S2-1: Hydrodynamic diameter of bPEG$_9$-AP205 VLP at varying molar ratio of BS(PEG)$_9$ to AP205 monomer.

Table S2-1: Summary of the measurements from Figure S2-1. Cumulant and Contin analysis algorithms were used for data analysis. D_h is the hydrodynamic diameter and PDI the polydispersity index.

Molar ratio	Cumulant D_h (nm)	PDI	Contin (1st peak) D_h (nm)
10x	35.0	0.10	38.8
20x	40.7	0.17	43.6
30x	37.1	0.14	42.0
40x	37.4	0.23	44.8
50x	40.0	0.18	45.6
100x	67.7	0.24	86.6

bPEG$_{25}$-AP205 VLP

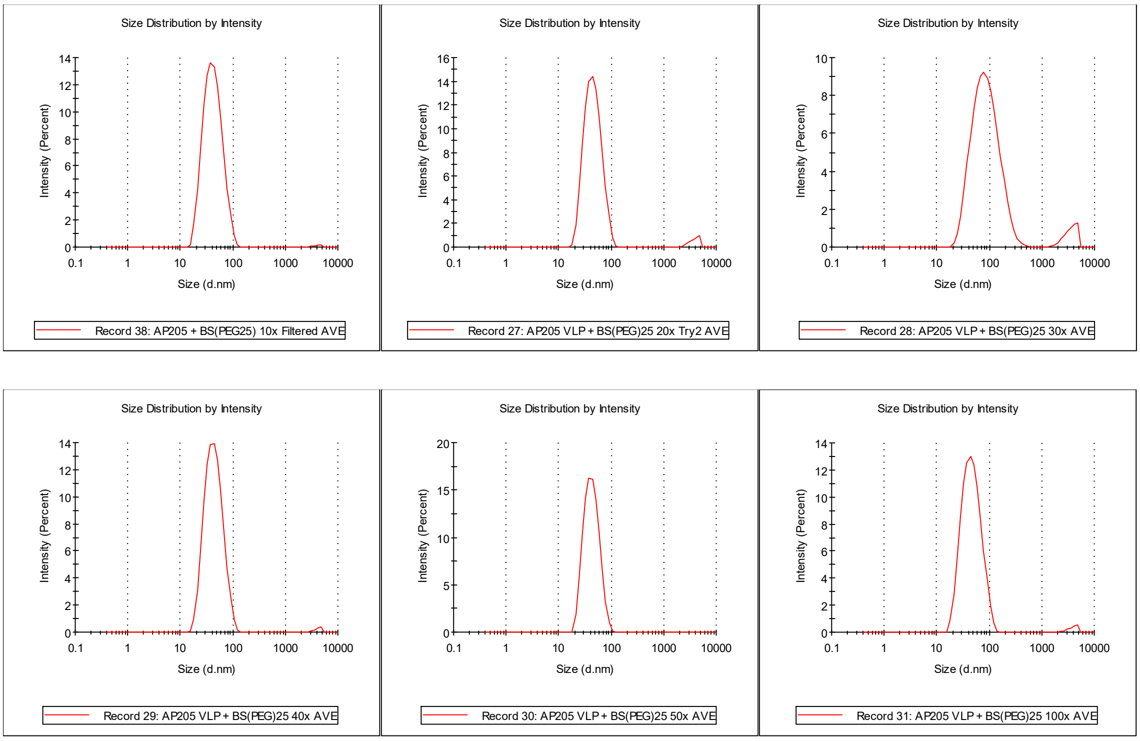

Figure S2-2: Hydrodynamic diameter of bPEG$_{25}$-AP205 VLP at varying molar ratio of BS(PEG)$_{25}$ to AP205 monomer.

Table S2-2: Summary of the measurements from Figure S2-2. Cumulant and Contin analysis algorithms were used for data analysis. D_{h} is the hydrodynamic diameter and PDI the polydispersity index.

Molar ratio	Cumulant $D_{\mathrm{h}}\ (\mathrm{nm})$	PDI	Contin (1st peak) $D_{\mathrm{h}}\ (\mathrm{nm})$
10x	38.8	0.16	44.2
20x	43.8	0.21	46.9
30x	86.0	0.21	97.6
40x	39.7	0.19	45.7
50x	39.8	0.13	43.9
100x	42.9	0.20	48.9

We finally emphasize that in our investigation of the crosslinking reaction, no aggregation was observed up to a VLP concentration of 4 mg/ml and a molar excess of the modifying PEG molecule to AP205 monomer of 10x, as determined by dynamic light scattering (DLS). It is important to note that this assertion assumes a low excess of dimethyl sulfoxide (DMSO), the organic solvent used to dissolve the PEG molecules. A low DMSO excess is achieved when a 10–100 mM PEG solution is used for the reaction. Conversely, when a 1.0 mM PEG solution is used, the excess of DMSO

increases by 10–100x, leading to partial aggregation of reaction products. Despite this drawback of using a 1.0 mM PEG solution, it is a potentially better PEG solution concentration due to easier handling and higher accuracy in maintaining the stoichiometry of the reaction, especially considering that our work involved very small VLP volumes (100–200 µl). After crosslinking, the product was thoroughly cleaned using ZebaSpin Desalting columns to remove excess PEG molecules. Subsequently, it was filtered by centrifugation (0.22 µm) to eliminate aggregates formed either due to DMSO excess or during ZebaSpin Desalting treatment.

S3. Morphology of PEG-crosslinked AP205 VLPs

Using transmission electron microscopy (TEM), we investigated the morphology of PEG-crosslinked AP205 VLPs. The images showed a spherical geometry after PEG-crosslinking with BS(PEG_9), BS(PEG_{13}), BS(PEG_{17}), BS(PEG_{21}) and BS(PEG_{25}).

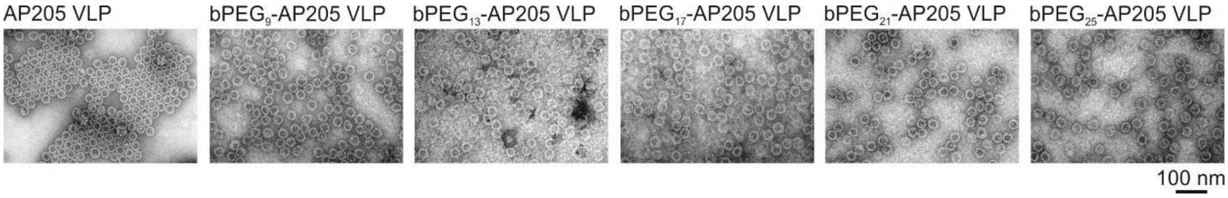

Figure S3-1: TEM images of native AP205 VLP, and bPEG_9-AP205 VLP, bPEG_{13}-AP205 VLP, bPEG_{17}-AP205 VLP, bPEG_{21}-AP205 VLP, bPEG_{25}-AP205 VLP.

S4. Characterization of PEG-crosslinked AP205 VLPs from the analysis of reducing SDS-PAGE

The gel shown in Fig. S5–1 (on the left) is reproduced from Fig. 1(c) in the main text. Densitometry profiles (on the right) were produced using ImageJ software for an indication of the AP205 dimers, trimers, and higher order oligomers as a result of the crosslinking reaction.

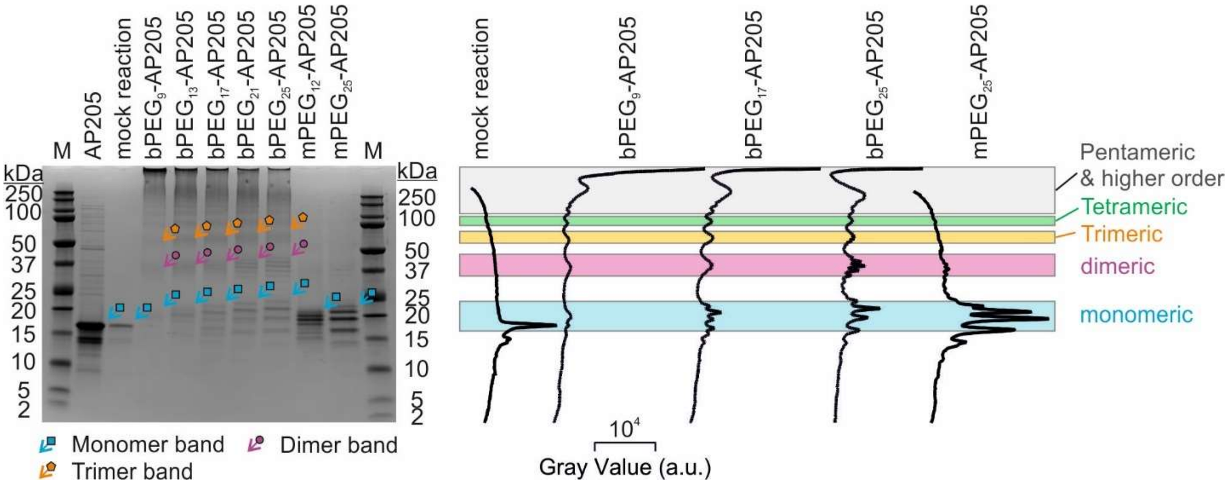

Figure S4-1: (Left–identical to Fig. 1(c) in the main text) Reducing SDS-PAGE of native AP205 VLP, AP205 VLP in mock reaction (i.e., in the presence of DMSO), PEG-crosslinked (bPEG$_n$-, n = 9–25), and PEGylated but not crosslinked AP205 VLPs (mPEG$_n$-, n = 12 or 25). M is PageRuler Plus Prestained Protein Ladder. The monomer region is marked with cyan arrows/squares, dimer region with purple arrows/circles and trimer region with orange arrows/pentagons. (Right) Densitometry profiles produced using ImageJ. The profiles clearly show the MW bands associated with AP205 dimers, trimers, tetramers, pentamers and higher order oligomers for the PEG-crosslinked AP205 VLPs. These bands were absent in the mock reaction and in mPEG$_{25}$-AP205 VLP.

The figure below shows the reducing SDS-PAGE results of AP205 VLP, AP205 VLP in mock reaction buffer, bPEG$_9$-AP205 VLP, bPEG$_5$-AP205 VLP, BS3-AP205 VLP and Sulfo-NHS-AP205 VLP. The VLPs bPEG$_9$-AP205, bPEG$_5$-AP205, BS3-AP205 and Sulfo-NHS-AP205 were prepared at three molar ratios, 1x, 5x and 10x. In lane 1, the MW band in the range 15–20 kDa corresponds to AP205 monomer. The same band appears in all the other lanes, expect that in lanes 5 and 6 versus 3 and lanes 7 and 8 versus 6, respectively for bPEG$_9$-AP205 VLP and bPEG$_5$-AP205 VLP, the bands were shifted to a higher molecular weight consistent with an increasing number of PEGylated AP205 monomers at higher molar ratio from mass spectroscopy. This statement does not apply to BS3-AP205 VLP and Sulfo-NHS-AP205 VLP which appear to be saturated already at 1x molar ratio. In cases of bPEG$_9$-AP205 VLP and bPEG$_5$-AP205 VLP, MW bands in the range

25–37 kDa appeared and corresponded to crosslinked AP205 dimers. Similarly, with $bPEG_9$-AP205 VLP and $bPEG_5$-AP205 VLP, the MW bands around 50 kDa appeared and corresponded to AP205 trimers. Bands corresponding to higher order oligomers were apparent for these VLPs. Unlike PEG-crosslinked AP205 VLPs, in lanes associated with BS3-AP205 VLP bands only in the dimeric region appeared while with Sulfo-NHS the band strength in the dimeric regions is similar to the native AP205 VLP.

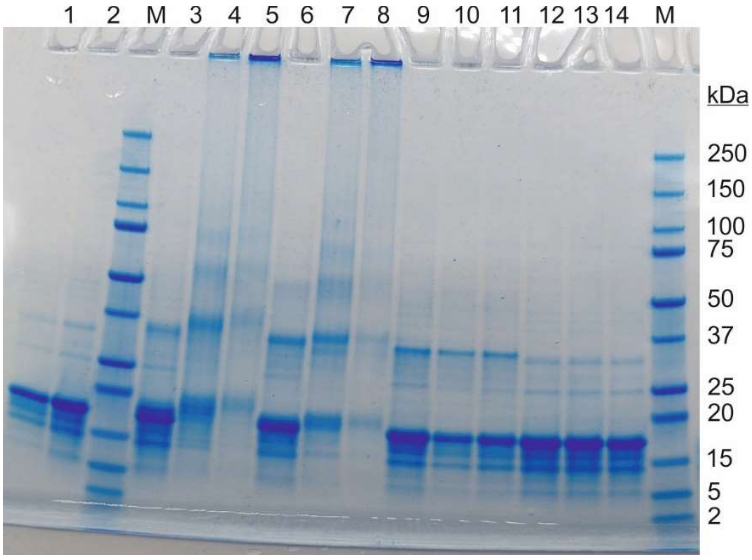

Figure S4-2: Reducing and denaturing SDS-PAGE of AP205 VLP (lane 1), AP205 VLP in mock reaction buffer (lane 2), $bPEG_9$-AP205 VLP at three different molar ratios 1x, 5x and 10x (lanes 3–5 respectively), $bPEG_5$-AP205 VLP at molar ratios 1x, 5x and 10x (lanes 6–8 respectively), BS3-AP205 VLP at molar ratios 1x, 5x and 10x (lanes 9–11 respectively) and Sulfo-NHS-AP205 VLP at molar ratios 1x, 5x and 10x (lanes 12–14 respectively).

The figure below shows reducing SDS-PAGE results of AP205 VLP, AP205 VLP in mock reaction buffer, $bPEG_5$-AP205 VLP, $bPEG_9$-AP205 VLP, $bPEG_{13}$-AP205 VLP, $bPEG_{17}$-AP205 VLP, $bPEG_{21}$-AP205 VLP, and $bPEG_{25}$-AP205 VLP. PEG-crosslinked AP205 VLPs were prepared at 1x molar ratio. The coat proteins of PEG-crosslinked VLPs showed a higher MW compared to AP205 coat protein monomer and the MW gradually increased with the length of the PEG. For $bPEG_{17}$-AP205 VLP (lane 6), $bPEG_{21}$-AP205 VLP (lane 7) and $bPEG_{25}$-AP205 VLP (lane 8), three to four bands were apparent in the monomeric MW range (15–25 kDa) that corresponded to singly, doubly, triply, and quadruple PEG-conjugated AP205 monomers . MW bands in the dimeric range (25–37 kDa) appeared for the PEG-crosslinked AP205 VLPs. Again, a clear graduation of PEG-crosslinked AP205 dimers bands corresponded to the MW of PEG crosslinkers. A dimer band was absent for AP205 VLP in lanes 1 and 2. PEG-crosslinked AP205 trimers and higher order oligomers were also apparent on the gel.

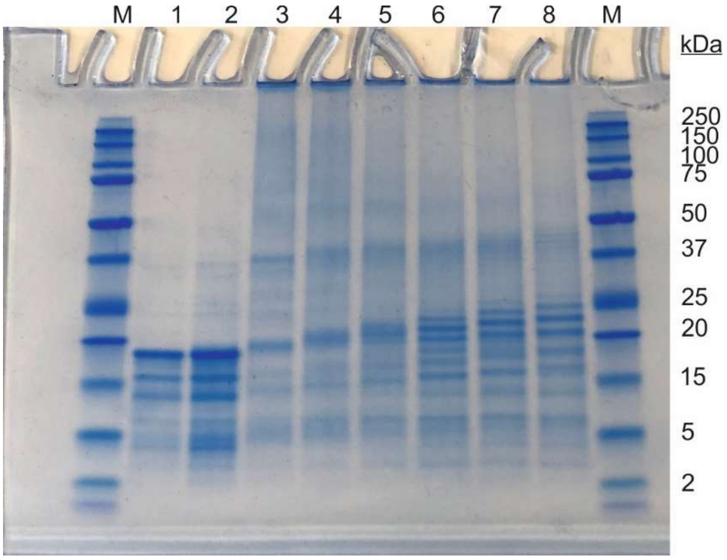

Figure S4-3: Reducing SDS-PAGE of AP205 (lane 1), AP205 in mock reaction buffer (lane 2), $bPEG_5$-AP205 VLP (lane 3), $bPEG_9$-AP205 VLP (lane 4), $bPEG_{13}$-AP205 VLP (lane 5), $bPEG_{17}$-AP205 VLP (lane 6), $bPEG_{21}$-AP205 VLP (lane 7) and $bPEG_{25}$-AP205 VLP (lane 8). All preparations were at 10x molar ratio.

S5. Mass spectrometric characterization of PEG-crosslinked AP205 VLPs

Electrospray ionization mass spectroscopy (ESI-MS) – "AP205 monomeric region"

From these measurements, the average MW of AP205 monomer was measured to be 15768 Da.

Reaction with BS(PEG_9) at 1x molar ratio resulted in a fraction of AP205 monomers remaining either native (i.e., no PEG molecule was conjugated to them), or conjugated to one PEG molecule. Increasing the molar ratio to 5x decreased the population of native AP205 monomers, while peaks at doubly- and triply-PEGylated AP205 monomers appeared. At 10x molar ratio, there was no population of native AP205 monomers, and we found a maximum of four PEG molecules per AP205 monomer in the monomeric region.

The variation of average MW between the individual peaks corresponded to the MW of one-end conjugated BS(PEG_9).

The reactions with Bis-dPEG_{13}-NHS ester, Bis-dPEG_{17}-NHS ester and Bis-dPEG_{25}-NHS ester followed a similar behavior; however, at 10x molar ratio, a maximum of three PEG molecules per AP205 monomer was detected.

ESI-MS of AP205 VLP

In the figure below, the mass spectrum of AP205 VLP is divided into three regions corresponding to the molecular weight of AP205 monomer (MW 15768 Da), dimer (MW 31536 Da), and trimer (MW 47304) which are indicated on each subfigure (respectively, left, middle and right).

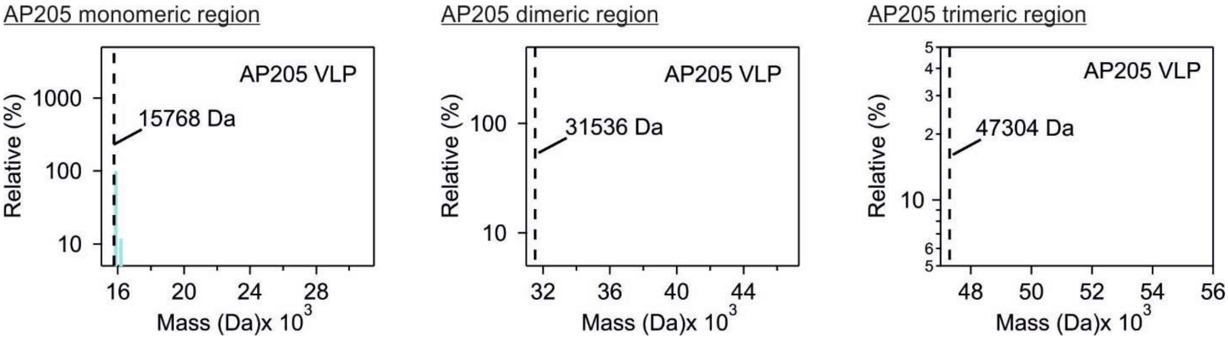

Figure S5-1: ESI-MS data of AP205 VLP.

ESI-MS of PEG-crosslinked AP205 VLPs

In the figures below, the mass spectra of PEG-crosslinked AP205 VLPs are shown. The spectra are divided into three regions corresponding to the molecular weight of AP205 monomer (MW

15768 Da), dimer (MW 31536 Da), and trimer (MW 47304 Da) which are indicated on each subfigure (respectively, left, middle and right). The tables following the figures summarize the occurrence of peaks in the “monomeric region”. The entries, only if they are highlighted in green, show the occurrence of native AP205 monomer and the occurrence of PEGylated AP205 monomers with 1 to 8 PEG molecules. The percentage abundance of each form, relative to the occurrence of the most populated form (= 100%), are indicated below the molecular weights. Only peaks above 10% occurrence are indicated in the tables.

ESI-MS of bPEG$_9$-AP205 VLP

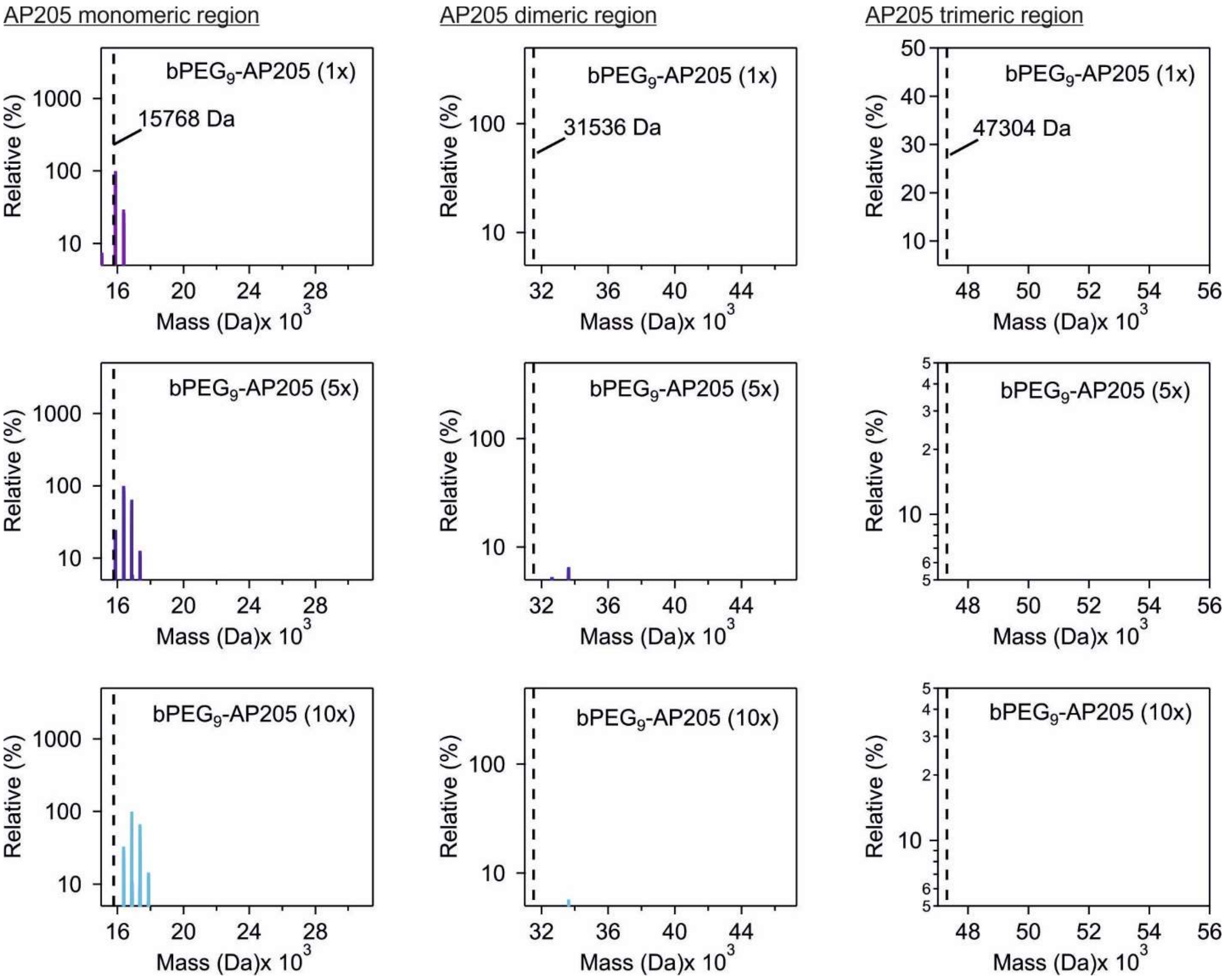

Figure S5-2: ESI-MS data of bPEG$_9$-AP205 VLP.

Table S5-1: Summary of the number and relative abundance of PEG$_9$-conjugated AP205 monomers.

Virus-like particle	Molar ratio[1]	15768 Da + 0x MW of crosslinker	15768 Da + 1x MW of crosslinker	15768 Da + 2x MW of crosslinker	15768 Da + 3x MW of crosslinker	15768 Da + 4x MW of crosslinker	15768 Da + 5x MW of crosslinker	15768 Da + 6x MW of crosslinker	15768 Da + 7x MW of crosslinker	15768 Da + 8x MW of crosslinker
bPEG$_9$-AP205	1x	15768 Da (100%)	16265 Da (29.5%)							
bPEG$_9$-AP205	5x	15768 Da (24.5%)	16265 Da (100%)	16761 Da (64.3%)	17258 Da (12.7%)					
bPEG$_9$-AP205	10x		16264 Da (33.1%)	16761 Da (100%)	17257 Da (67.4%)	17772 Da (14.6%)				

[1] [molecule/AP205 monomer]

ESI-MS of bPEG$_{13}$-AP205 VLP

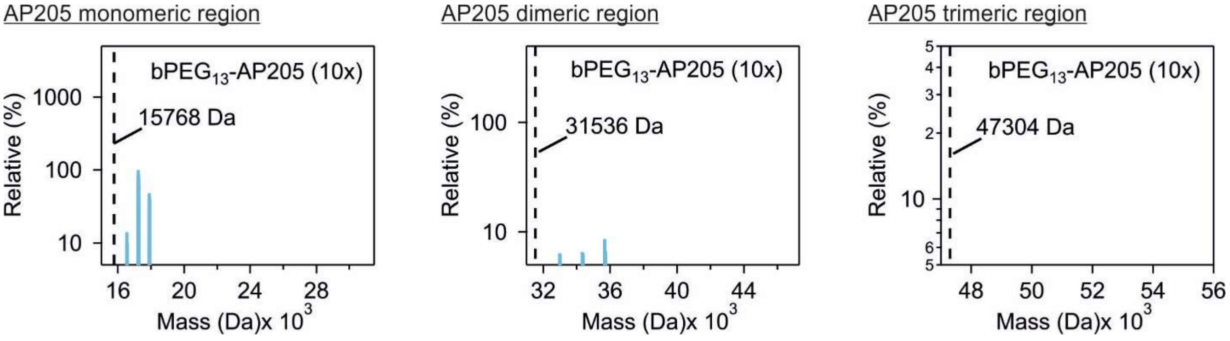

Figure S5-3: ESI-MS data of bPEG$_{13}$-AP205 VLP.

Table S5-2: Summary of the number and relative abundance of PEG$_{13}$-conjugated AP205 monomers.

Virus-like particle	Molar ratio[1]	15768 Da + 0x MW of crosslinker	15768 Da + 1x MW of crosslinker	15768 Da + 2x MW of crosslinker	15768 Da + 3x MW of crosslinker	15768 Da + 4x MW of crosslinker	15768 Da + 5x MW of crosslinker	15768 Da + 6x MW of crosslinker	15768 Da + 7x MW of crosslinker	15768 Da + 8x MW of crosslinker
bPEG$_{13}$-AP205	10x		16441 Da (14.2%)	17113 Da (100%)	17786 Da (48.2%)					

[1] [molecule/AP205 monomer]

ESI-MS of bPEG$_{17}$-AP205 VLP

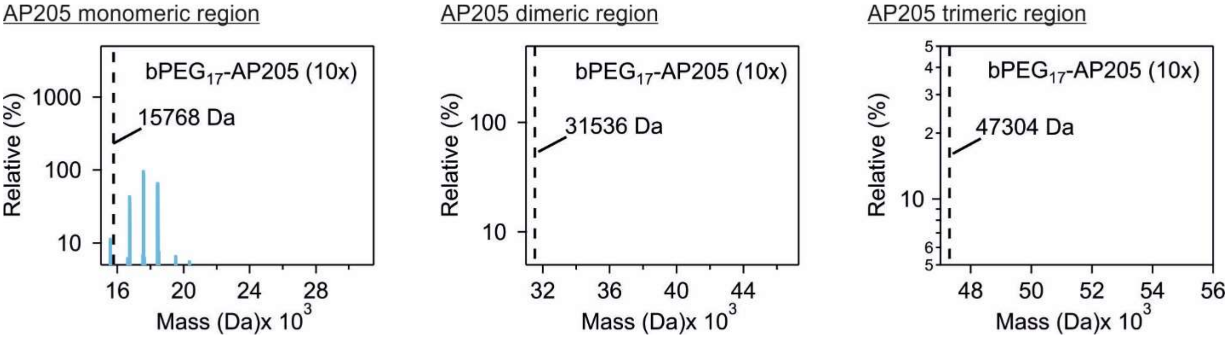

Figure S5-4: ESI-MS data of bPEG$_{17}$-AP205 VLP.

Table S5-3: Summary of the number and relative abundance of PEG$_{17}$-conjugated AP205 monomers.

Virus-like particle	Molar ratio[1]	15768 Da + 0x MW of crosslinker	15768 Da + 1x MW of crosslinker	15768 Da + 2x MW of crosslinker	15768 Da + 3x MW of crosslinker	15768 Da + 4x MW of crosslinker	15768 Da + 5x MW of crosslinker	15768 Da + 6x MW of crosslinker	15768 Da + 7x MW of crosslinker	15768 Da + 8x MW of crosslinker
bPEG$_{17}$-AP205	10x		16617 Da (45.1%)	17466 Da (100%)	18332 Da (68.9%)					

[1] [molecule/AP205 monomer]

ESI-MS of bPEG$_{21}$-AP205 VLP

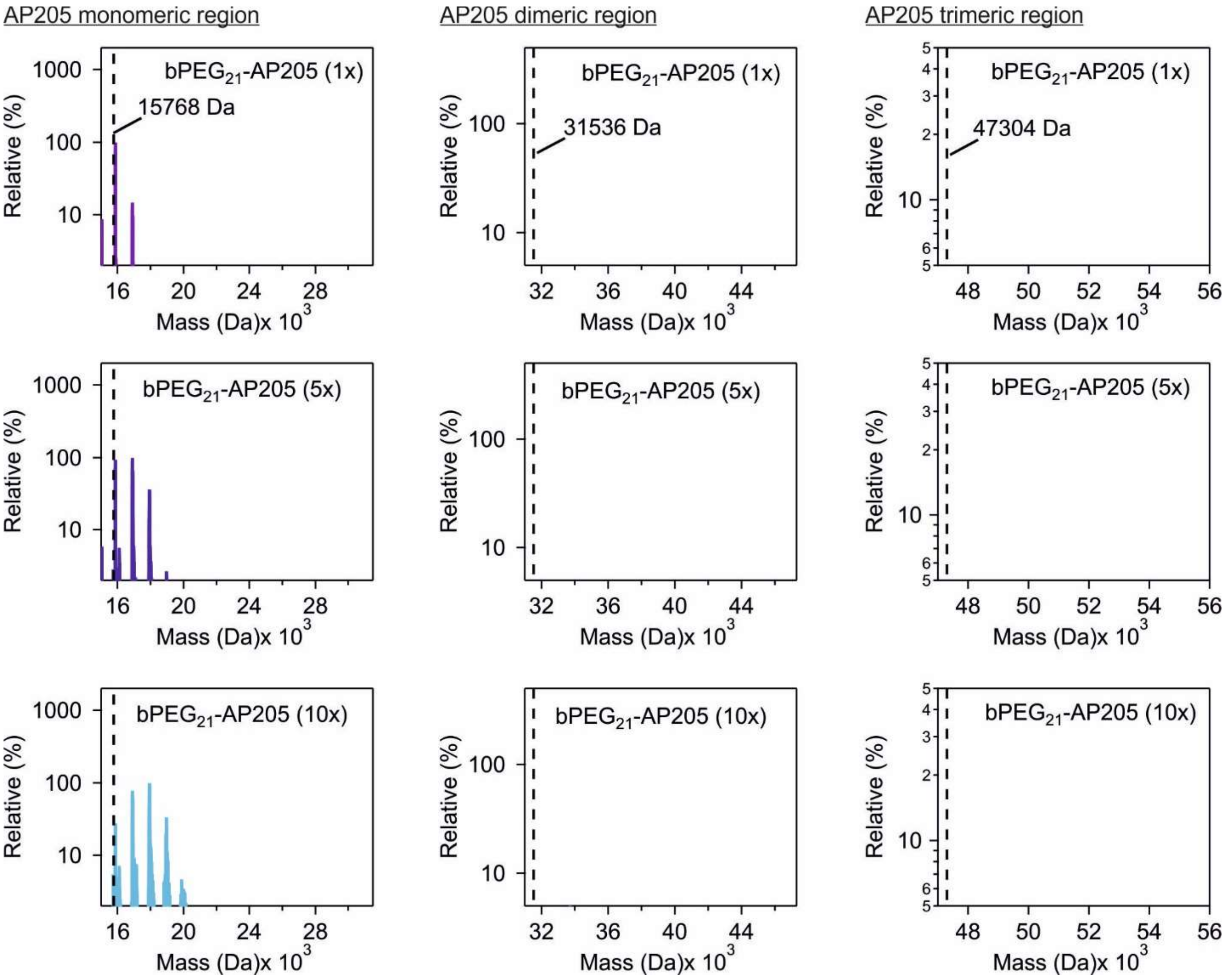

Figure S5-5: ESI-MS data of bPEG$_{21}$-AP205 VLP.

Table S5-4: Summary of the number and relative abundance of PEG$_{21}$-conjugated AP205 monomers.

Virus-like particle	Molar ratio[1]	15768 Da + 0x MW of crosslinker	15768 Da + 1x MW of crosslinker	15768 Da + 2x MW of crosslinker	15768 Da + 3x MW of crosslinker	15768 Da + 4x MW of crosslinker	15768 Da + 5x MW of crosslinker	15768 Da + 6x MW of crosslinker	15768 Da + 7x MW of crosslinker	15768 Da + 8x MW of crosslinker
bPEG$_{21}$-AP205	1x	15768 Da (100%)	16793 Da (14.8%)							
bPEG$_{21}$-AP205	5x	15768 Da (94.3%)	16793 Da (100%)	17835 Da (36.7%)						
bPEG$_{21}$-AP205	10x	15768 Da (27.8%)	16793 Da (78%)	17836 Da (100%)	18861 Da (33.4%)					

[1] [molecule/AP205 monomer]

ESI-MS of bPEG$_{25}$-AP205 VLP

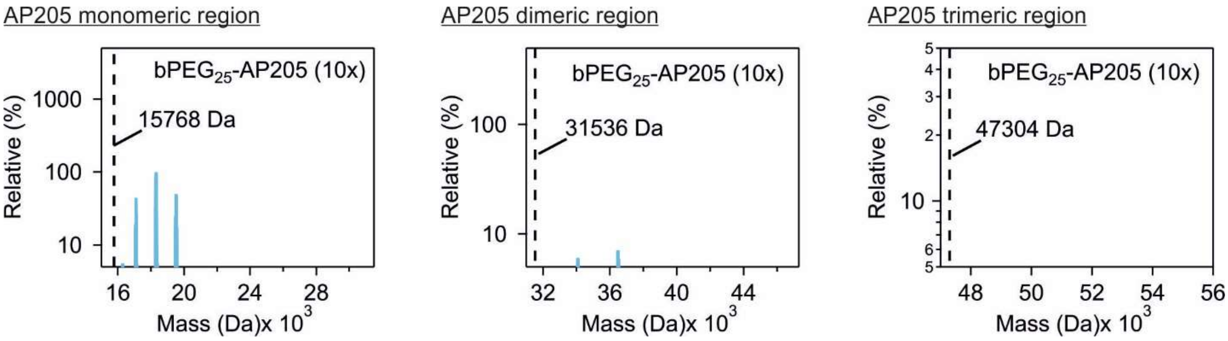

Figure S5-6: ESI-MS data of bPEG$_{25}$-AP205 VLP.

Table S5-5: Summary of the number and relative abundance of PEG$_{25}$-conjugated AP205 monomers.

Virus-like particle	Molar ratio[1]	15768 Da + 0x MW of crosslinker	15768 Da + 1x MW of crosslinker	15768 Da + 2x MW of crosslinker	15768 Da + 3x MW of crosslinker	15768 Da + 4x MW of crosslinker	15768 Da + 5x MW of crosslinker	15768 Da + 6x MW of crosslinker	15768 Da + 7x MW of crosslinker	15768 Da + 8x MW of crosslinker
bPEG$_{25}$-AP205	10x		16987 Da (44.5%)	18205 Da (100%)	19406 Da (50.3%)					

[1] [molecule/AP205 monomer]

Nano ultra-performance liquid chromatography coupled to mass spectroscopy (NanoUPLC-MS/MS) analysis of bPEG$_9$-AP205 VLP – "AP205 dimeric region"

The results showed that five out of seven lysine residues together with the N-terminus were modified by one-end conjugated BS(PEG$_9$) with the molecular formula $C_{22}O_{12}H_{40}$ (MW 496.3 Da) (Table S5-6).

Furthermore, two "intra"-AP205 monomer linkages between the N-terminus (A2) and lysine 4 (K4) and between K94 and K101 were detected.

In agreement with the results of reducing SDS-PAGE, the LC-MS/MS analysis revealed the presence of "inter"-AP205 monomer crosslinking. In this case, two AP205 monomers were connected by PEG$_9$ crosslinker with the molecular formula $C_{22}O_{11}H_{38}$ (MW 478.3 Da). The cross linkages were between K4 and K61 as well as between K4 and K94. More details in below:

The raw data were analyzed by Byonic 4.0 (Protein Metrics, USA) with the consideration of mass tolerance of 5 ppm at MS1 level, and 20 ppm at MS2 level and a score higher than 200. To confirm the results, Spectrum Identification Machine (SIM-XL) was also used for crosslink analysis [1].

For crosslinked peptides, the results from SIM-XL showed that there were two intra-linked peptides at A2-K4 and K94-K101 (Figure S5-7 and Table S5-7). In addition, two cross-linked peptides were observed, namely A2/K4-K61 and A2/K4-K94. The software did not confirm either N-terminus A2 or lysine K4 participated in the crosslinking reaction with the other lysine residue (K61 or K94) due to the lack of key ions. However, if the major linkage was between N-terminus and K61 or K94, K4 residue would have been cleaved off by trypsin. Then, the results would have shown a linkage between ^{2}AN^{4}K-crosslinked with the partner peptide. But the results showed ^{2}AN^{4}KPMQPITSTAN^{15}K linked to the other peptide. Therefore, the data suggested that most likely, K4 instead of A2 was linked to the other lysine. Byonic results agreed with the assignment from the SIM-XL software.

The location of the afore-mentioned N-terminus and lysine residues are noted in the sequence below:

M.^{2}AN^{4}KPMQPITSTAN^{15}KIVWSDPTRLSTTFSASLLRQRV^{38}KVGIAELNNVSGQYVSVY^{56}KRPA
P^{61}KPEGCADACVIMPNENQSIRTVISGSAENLATL^{94}KAEWETH^{101}KRNVDTLFASGNAGLGFLD
PTAAIVSSDTTAGSGGAHATANATAHHHHHH

Table S5-6: Summary of Byonic analysis of bPEG9-AP205 VLP based on the following criteria: score higher than 200 and peptides correctly cleaved at Lys and Arg residues.

Position	Tryptic peptides	Observed linker[1]	Score	PSM
2 or 4	M.[2]AN[4]KPMQPITSTANK.I[2]	K4(+$C_{22}O_{12}H_{40}$)	797.2	111
15	M.AN[4]KPMQPITSTAN[15]KIVWSDPTR.L	K4(+$C_{22}O_{12}H_{40}$) K15 (+$C_{22}O_{12}H_{40}$)	885.3	5
38	R.V[38]KVGIAELNNVSGQYVSVYK.R	K38(+$C_{22}O_{12}H_{40}$)	954.7	14
56	R.VKVGIAELNNVSGQYVSVY[56]K.R	--[3]	-	-
61	R.PAP[61]KPEGCADACVIMPNENQSIR.T	K61(+$C_{22}O_{12}H_{40}$)	511.8	6
94	R.TVISGSAENLATL[94]K.A	K94(+$C_{22}O_{12}H_{40}$)	534.7	2
101	R.TVISGSAENLATLKAEWETH[101]K.R	---[3]	-	-

Note:

1. The chemical format for one-end conjugated PEG9 adduct on lysine is $C_{22}O_{12}H_{40}$ and the exact mass is 496.26. For PEG9-crosslinked peptides, the mass is the sum of two peptides plus 478.26 ($C_{22}O_{11}H_{38}$).
2. The MS/MS spectra could not confirm which amino acid was modified by PEG9 due to the lack of b1 and b2 ions.
3. The lysine was not labeled with PEG9.

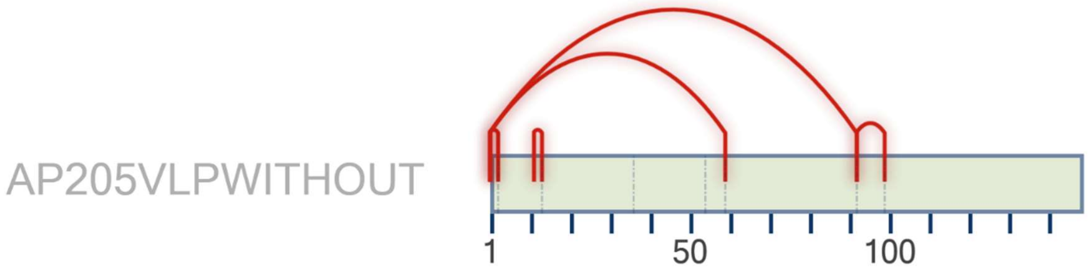

Figure S5-7: The scheme of crosslinked peptides from bPEG9-AP205 VLP via SIM search engine. The fasta sequence that was used to perform the search started from A at the N-terminus. Therefore, the position of modified amino acids had 1 position less than the full sequence. The identified peptides are summarized in Table 2.

Table S5-7: Summary of PEG$_9$-cross-linked peptides from SIM-XL search engine

Intra-linked

Scan Number	Experimental M+H	Type Peptide Link	Primary Score	ppm	Peaks Matched	Peptide Sequence	Position of XL-Residue 1	Position of XL-Residue 2
14584	1979.02355							
		Intra	2.97394	8.78	25	ANKPMQPITSTANK	1	3
14580	1979.022975							
		Intra	2.81724	9.071	19	ANKPMQPITSTANK	1	3
14364	1979.02475							
		Intra	2.72725	8.174	23	ANKPMQPITSTANK	1	3
19553	2919.51845							
		Intra	2.25529	5.779	18	TVISGSAENLATLKAEWETHKR	93	100

Inter-linked

Scan Number	Experimental M+H	Type Peptide Link	Primary Score	ppm	Peaks Matched	Peaks Matched ß	Peptide Sequence	Position of XL-Residue 1	Position of XL-Residue 2
16148	4419.294875								
		Inter	5.59702	3.858	33	21	TVISGSAENLATLKAEWETHKR - ANKPMQPITSTANK	93	1
		Inter	5.49283	3.858	33	20	TVISGSAENLATLKAEWETHKR - ANKPMQPITSTANK	93	3
12054	4688.3032								
		Inter	4.60746	3.46	31	12	RPAPKPEGC(57.02146)ADAC(57.02146)VIMPNENQSIR - ANKPMQPITSTANK	60	1
		Inter	4.3861	3.46	31	10	RPAPKPEGC(57.02146)ADAC(57.02146)VIMPNENQSIR - ANKPMQPITSTANK	60	3
16128	4419.2897								
		Inter	3.85457	5.029	23	13	TVISGSAENLATLKAEWETHKR - ANKPMQPITSTANK	93	1
		Inter	3.63072	5.029	23	11	TVISGSAENLATLKAEWETHKR - ANKPMQPITSTANK	93	3
12055	4688.3032								
		Inter	3.49869	3.46	24	9	RPAPKPEGC(57.02146)ADAC(57.02146)VIMPNENQSIR - ANKPMQPITSTANK	60	1

S6. Investigation of colloidal stability

Using dynamic light scattering (DLS), the colloidal stability of native and PEG-crosslinked AP205 VLPs were investigated in phosphate buffered saline (PBS) at pH 7.4, and in acidified PBS, pH 2.0–6.0. The pH-adjusted PBS was prepared by adding an appropriate amount of 100 mM HCl to PBS. Thereafter, 10 µl of 1 mg/ml VLP solution was added to 90 µl of acidified PBS. Since the addition of VLP solution increased the pH of acidified PBS, especially in the cases of pH 4.0 and pH 5.0, acidified PBS were prepared at a slightly lower pH.

In summary, native AP205 VLP was stable at pH 7.4, but aggregated at pH values 2.0–6.0. In particular, already at pH 6.0, in the ‘size distribution by intensity’ spectrum, a peak associated with micron-size aggregates was observed. A pH dependent colloidal stability agrees with charge regulation of AP205 coat proteins.

The PEG-crosslinking of AP205 VLP increased the colloidal stability profile up to pH ~ 4.0. The stability was mediated by steric repulsion of surface-conjugated PEG molecules and not by electrostatics. This is because the reaction of lysine residue or N-terminus with functional PEG linker reduces the positive charge of these moieties.

Native AP205 VLP

The colloidal stability of AP205 VLP was narrow, e.g., at pH ≤ 6.0, the VLP was readily aggregated. At pH 2.0, the VLP was somewhat stable. Therefore, the stable pH for AP205 VLP in PBS is pH 7.4.

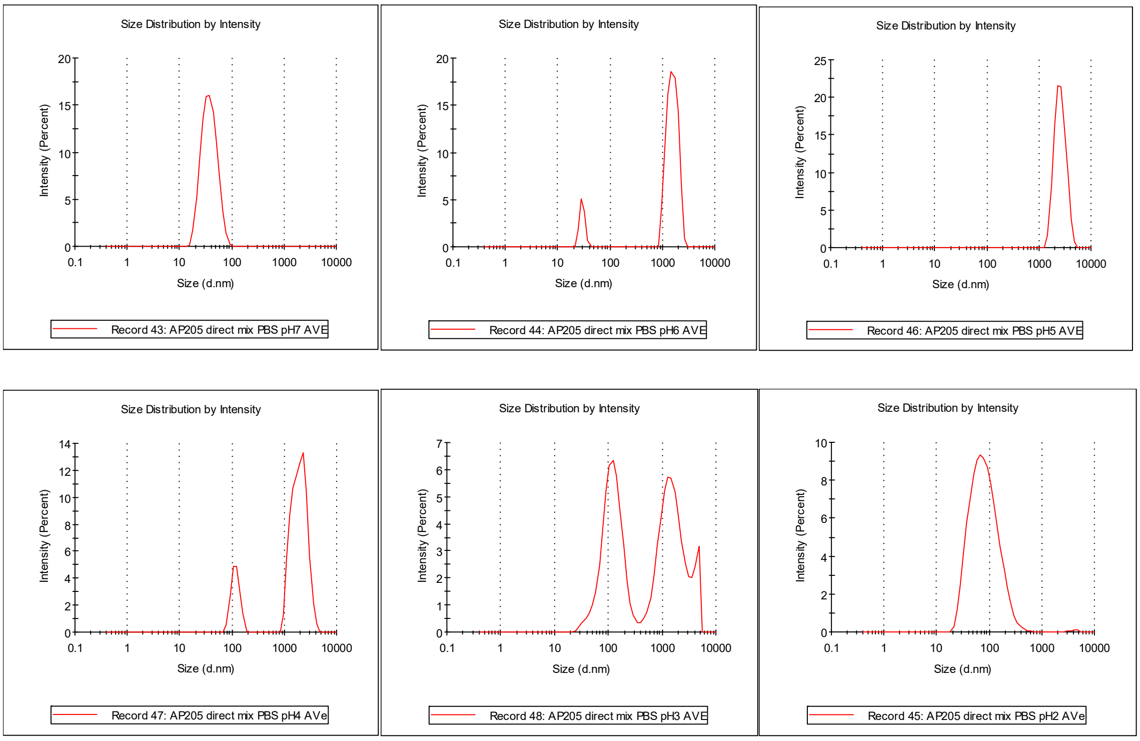

Figure S6-1: Hydrodynamic diameter of AP205 VLP at varying pH 7.4 to 2.0.

Table S6-1: Summary of the measurements from Figure S6-1. Cumulant and Contin analysis algorithms were used for data analysis. D_h is the hydrodynamic diameter, and PDI the polydispersity index.

pH	Cumulant D_h (nm)	PDI	Contin (1st peak) D_h (nm)
7.4	35.0	0.12	38.8
6.0	1721	0.73	29.7
5.0	2470	0.21	2613
4.0	1458	0.88	118.3
3.0	268.4	0.89	127.5
2.0	68.5	0.23	93.5

Comparison of colloidal stability between native and PEG-crosslinked AP205 VLPs

The figure below shows the effect of pH on colloidal stability of native and PEG-crosslinked AP205 VLPs. The data suggested that AP205 VLP was aggregated in pH 6.0–3.0. The origin of this aggregation is electrostatics. As a function of pH, the surface charge density of AP205 coat protein monomers varies leading to instability of the VLP. In the cases of bPEG$_9$-AP205 VLP and bPEG$_{21}$-AP205 VLP, the VLPs remained stable up to pH 4.0. Therefore, PEG-crosslinking increased the colloidal stability at low pH.

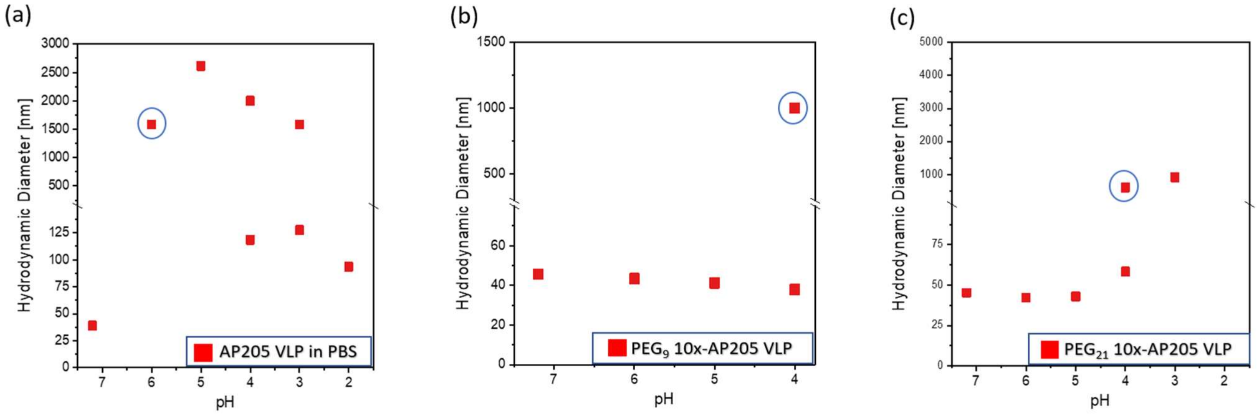

Figure S6-2: Hydrodynamic diameter of AP205 VLP, bPEG$_9$-AP205 VLP and bPEG$_{21}$-AP205 VLP at varying pH 7.4 to 2.0. Blue circles indicate the pH onset of aggregation from basic to acid solution.

S7. Investigation of enzymatic stability in gastric fluid

Table S7-1: Type and pH of gastric fluid used in the investigations of enzymatic stability of native and PEGylated AP205 VLPs.

Type	Animal number	pH
Pig gastric fluid	1314	3.0
Pig gastric fluid	1309	4.6
Pig gastric fluid	1257	5.5
Mouse gastric fluid	Pooled	4.6

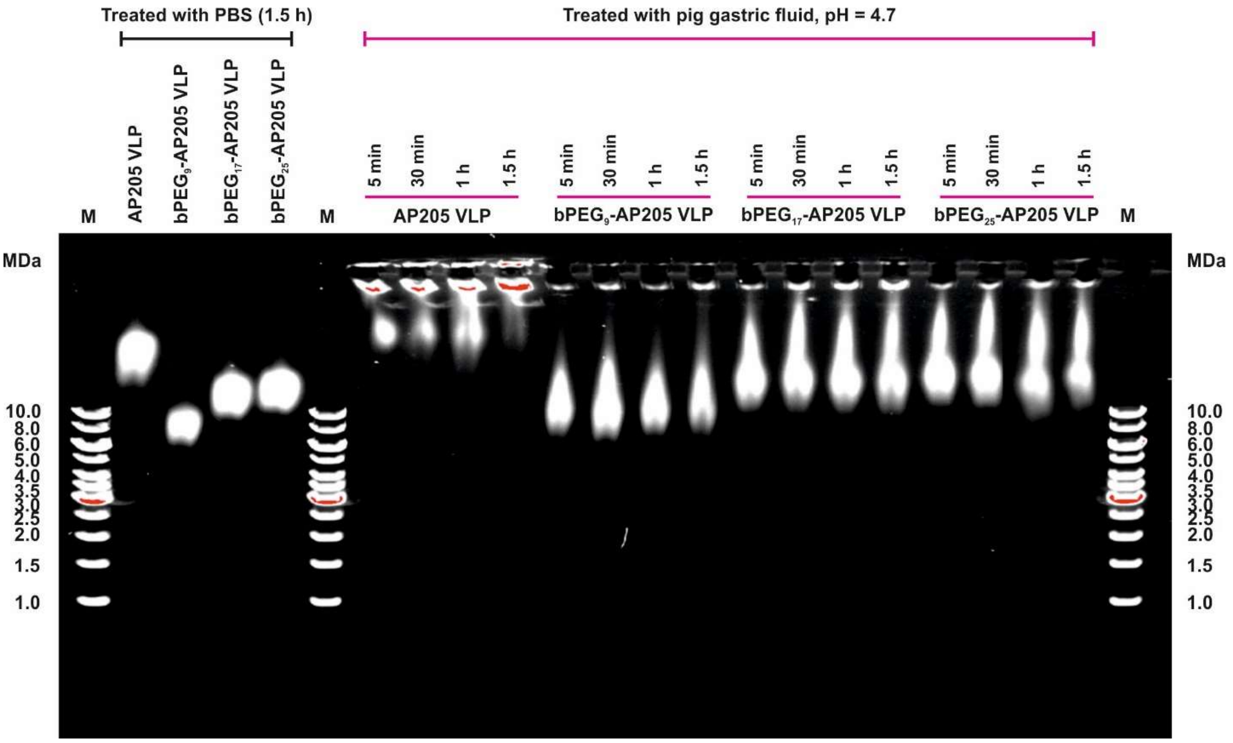

Figure S7-1. Enzymatic stability of native and PEGylated AP205 VLPs in pig gastric fluid at pH 4.7. Agarose gel of AP205 VLP, bPEG$_9$-AP205 VLP, bPEG$_{17}$-AP205 VLP and bPEG$_{25}$-AP205 VLP in PBS and in pig gastric fluid, respectively on the left and right of the gel. VLP incubation times included 5 min, 30 min, 1 hour and 1.5 hours at 37°C. M stands for marker.

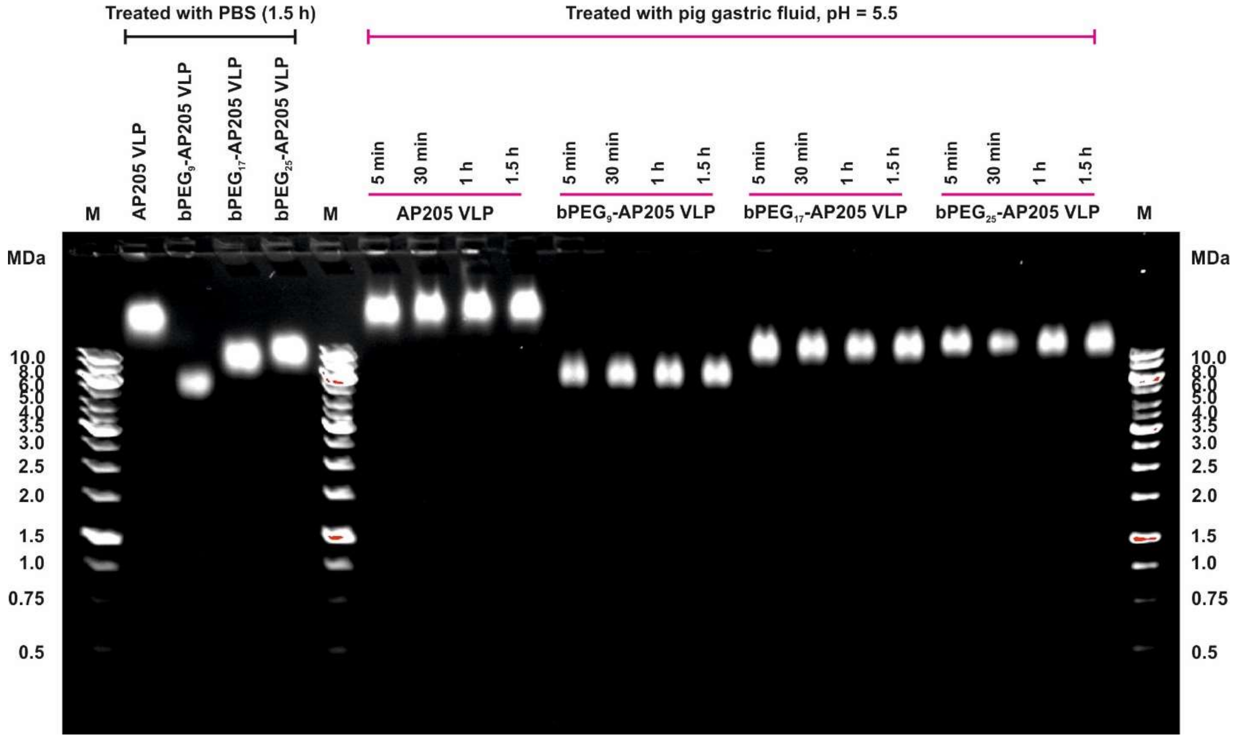

Figure S7-2. Enzymatic stability of native and PEGylated VLPs in pig gastric fluid at pH 5.5. Agarose gel of AP205 VLP, $bPEG_9$-AP205 VLP, $bPEG_{17}$-AP205 VLP and $bPEG_{25}$-AP205 VLP in PBS and in pig gastric fluid, respectively on the left and right of the gel. VLP incubation times included 5 min, 30 min, 1 hour and 1.5 hours at 37°C. M stands for marker.

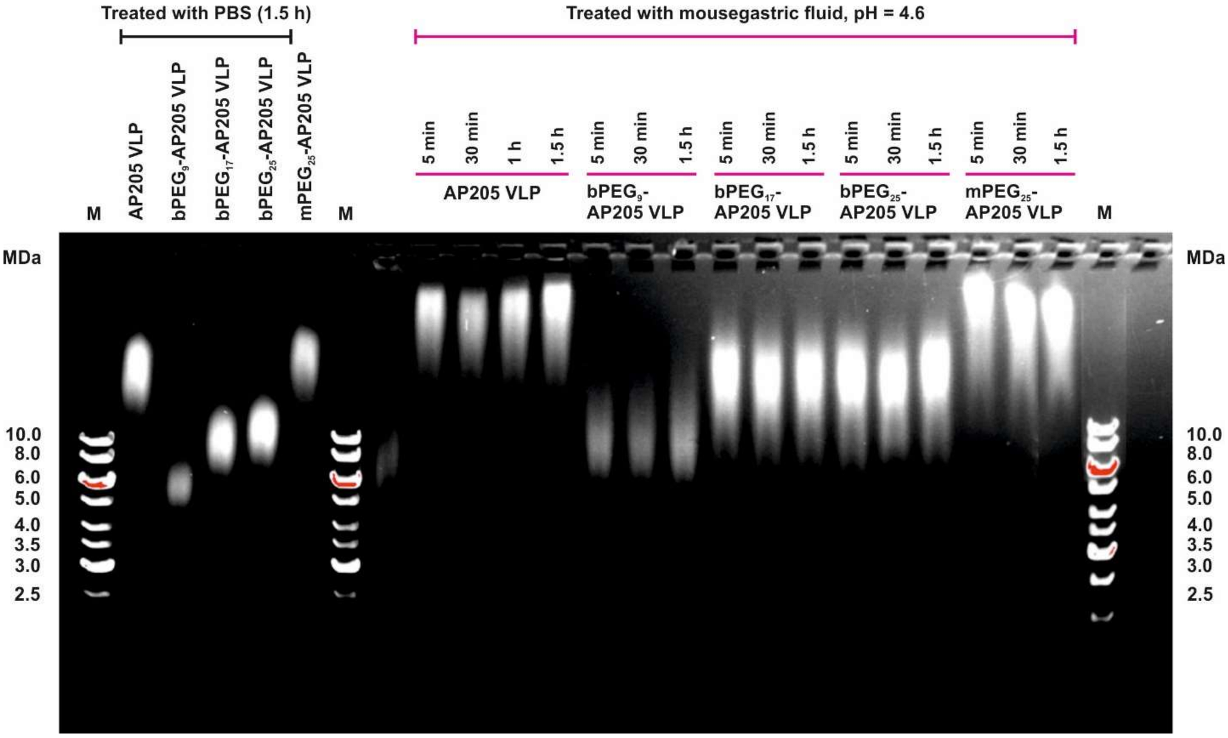

Figure S7-3. Enzymatic stability of native and PEGylated VLPs in mouse gastric fluid at pH 4.6. Agarose gel of AP205 VLP, $bPEG_9$-AP205 VLP, $bPEG_{17}$-AP205 VLP, $bPEG_{25}$-AP205 VLP and $mPEG_{25}$-AP205 VLP in PBS and in mouse gastric fluid, respectively on the left and right of the gel. VLP incubation times included 5 min, 30 min and 1.5 hours at 37°C. M stands for marker.

For semi-quantification of the degradation, after each time point, an aliquot was added to a solution of cOmplete™, EDTA-free Protease Inhibitor Cocktail and DNA Loading Dye, then placed on dry ice. The resulting agarose gel electrophoresis is shown below. The estimation of degradation rate was from the slope of gray value intensity with respect to time within the first 5 min. After 5 min, the background was too high for a precise gray value indication.

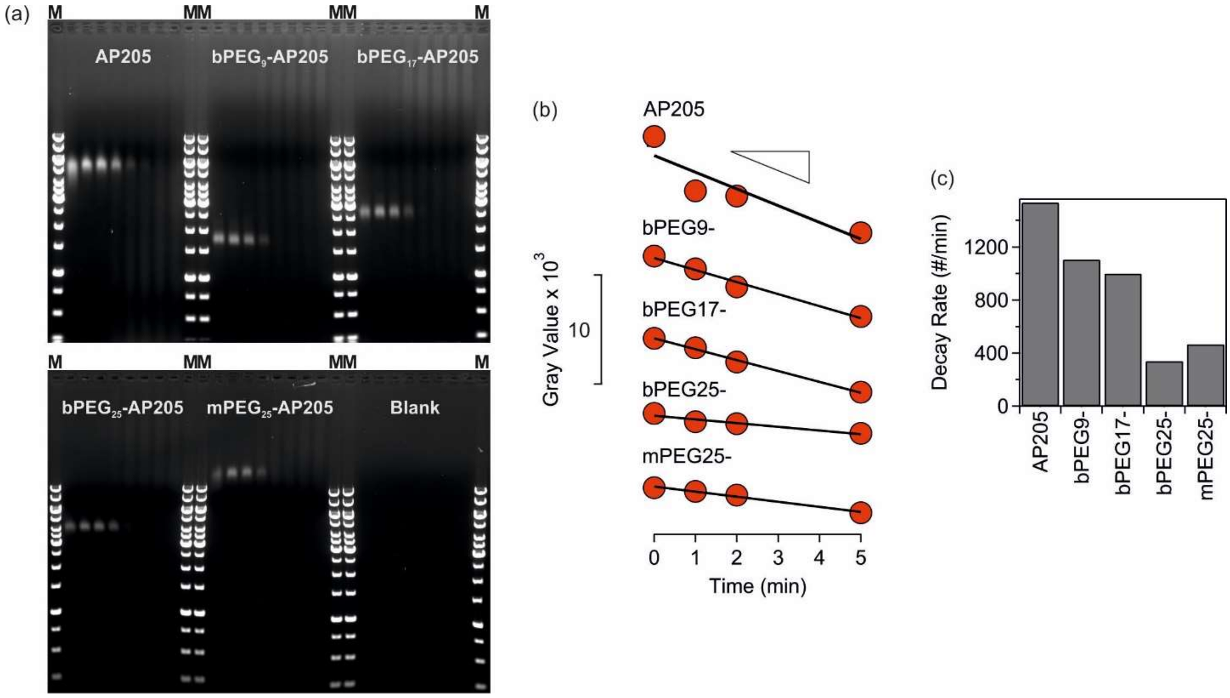

Figure S7-4. Enzymatic stability of native and PEGylated VLPs in pig gastric fluid at pH 3.0. (a) Agarose gel of AP205 VLP, $bPEG_9$-AP205 VLP, $bPEG_{17}$-AP205 VLP, $bPEG_{25}$-AP205 VLP and $mPEG_{25}$-AP205 VLP incubated with pig gastric fluid (pH 3.0) at time points 0 min, 1 min, 2 min, 5 min, 15 min, 30 min, 60 min and 120 min in consecutive lanes. M stands for marker. (b) Reduction in gray value intensity (calculated using ImageJ) as a function of time in the period 0–5 min. (c) Degradation rate estimated from the slop of the reduction in gray value with respect to time.

S8. Translocation of native and PEGylated AP205 VLPs through mucus in *in vitro* 3D human nasal epithelial tissues

Results of VLP delivery to tissues 1 and 2

Figure S8-1 shows the accumulation height and the mean fluorescence intensity (MFI) of the translocated native and PEG-crosslinked AP205 VLPs on tissue 1 (MD0860) and tissue 2 (MD0871). On each tissue, a similar accumulation height between the native AP205 VLP and the PEG-crosslinked AP205 VLPs was expected. The general behavior shows that the VLPs behaved somewhat similarly in terms of mucus translocation.

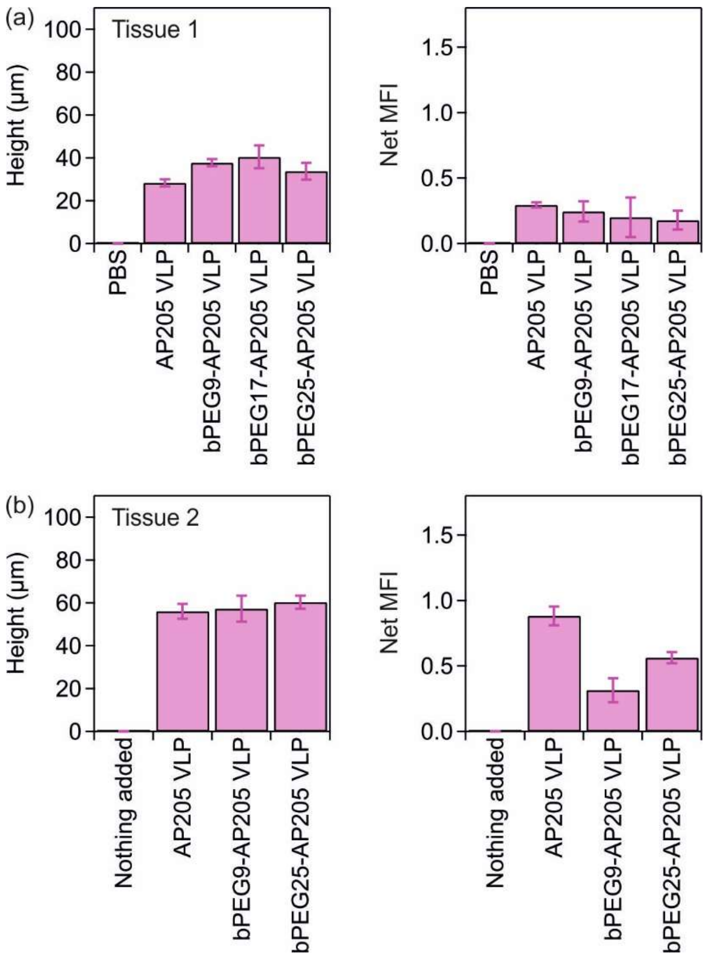

Figure S8-1: The accumulation height and the mean fluorescence intensity (MFI) of the translocated native and PEG-crosslinked AP205 VLPs on nasal monodonor tissue 1 (MD0860) (a) and on nasal monodonor tissue 2 (MD0871) (b). In the experiments with tissue 1, PBS was added as a control. In the experiments with tissue 2, no liquid was added as control.

Effect of mucociliary clearance on inhibition on lateral distribution and vertical translocation of VLPs

Additional figures on the effect of ciliary beating and mucociliary clearance on lateral distribution and vertical translocation of delivered particles are shown below. In these experiments, tissue 3 (MD0774) was used.

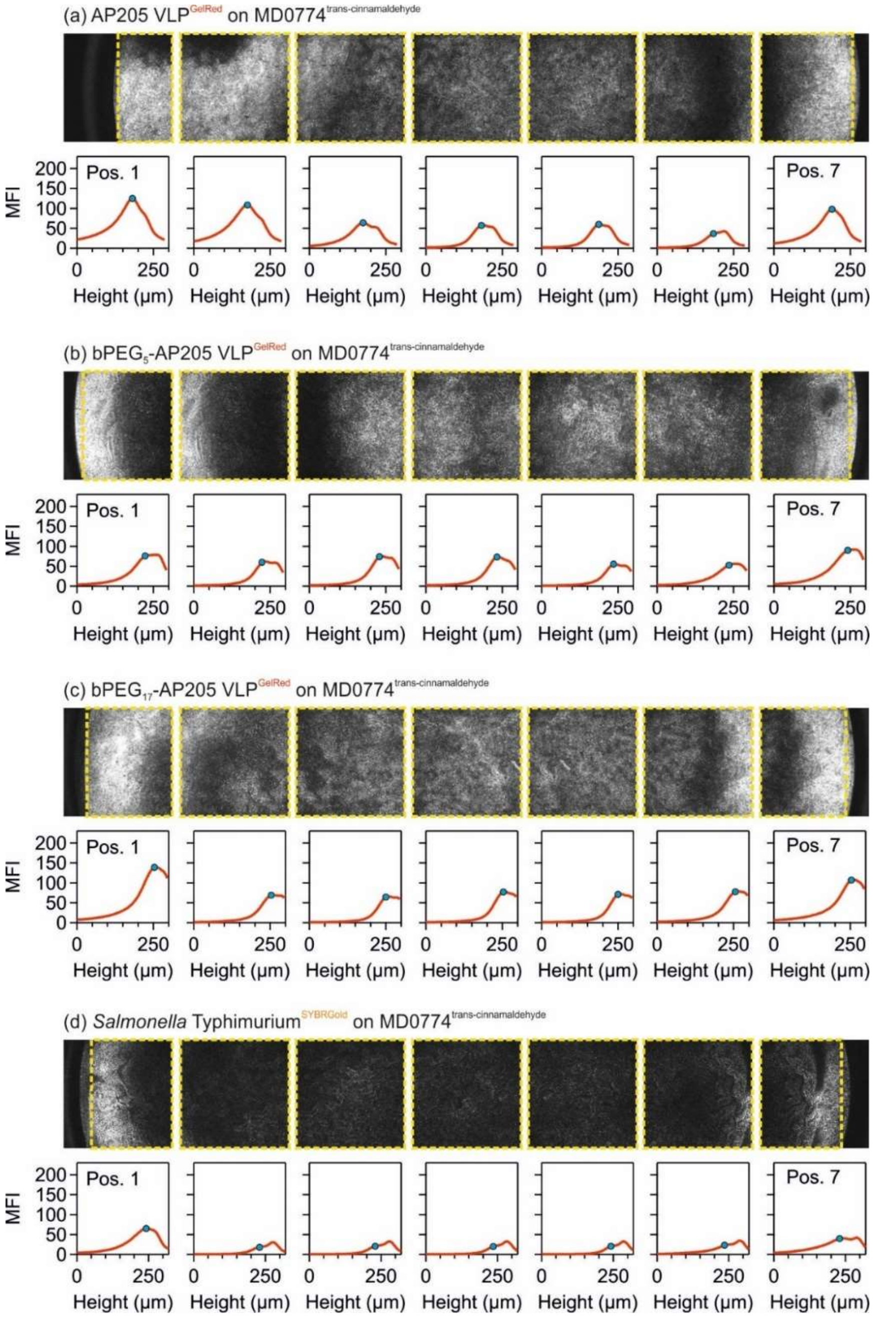

Figure S8-2. Effect of ciliary beating on distribution of delivered VLPs. Delivery of (a) AP205 VLP, (b) bPEG$_5$-AP205 VLP, (c) bPEG$_{17}$-AP205 VLP and (d) *Salmonella* Typhimurium to tissue 3 (MD0774) in the presence of ciliary beating inhibitory molecule trans-cinnamaldehyde. The yellow

box denotes the area of mean fluorescence intensity (MFI) calculation and blue circle the location of VLP accumulation. In these figures, height starts from above the air-liquid interface (ALI) and increases in the direction of cell layer to below the membrane.

References

(1) Lima, D. B.; de Lima, T. B.; Balbuena, T. S.; Neves-Ferreira, A. G. C.; Barbosa, V. C.; Gozzo, F. C.; Carvalho, P. C. Sim-Xl: A Powerful and User-Friendly Tool for Peptide Cross-Linking Analysis. *Journal of Proteomics*. **2015**, *129*, 51-55.
